# Social media enables people-centric climate action in the hard-to-decarbonise building sector

**DOI:** 10.1038/s41598-022-23624-9

**Published:** 2022-11-17

**Authors:** Ramit Debnath, Ronita Bardhan, Darshil U. Shah, Kamiar Mohaddes, Michael H. Ramage, R. Michael Alvarez, Benjamin K. Sovacool

**Affiliations:** 1 University of Cambridge, Cambridge, CB2 1TN United Kingdom; 2 California Institute of Technology, Pasadena, CA 91125 United States; 3 Boston University, Boston, MA 02215 United States; 4 University of Sussex Business School, Brighton, BN1 9SN United Kingdom; 5 Aarhus University, Aarhus, 8000 Denmark

**Keywords:** Climate-change policy, Environmental social sciences, Sustainability

## Abstract

The building and construction sector accounts for around 39% of global carbon dioxide emissions and remains a hard-to-abate sector. We use a data-driven analysis of global high-level climate action on emissions reduction in the building sector using 256,717 English-language tweets across a 13-year time frame (2009–2021). Using natural language processing and network analysis, we show that public sentiments and emotions on social media are reactive to these climate policy actions. Between 2009–2012, discussions around green building-led emission reduction efforts were highly influential in shaping the online public perceptions of climate action. From 2013 to 2016, communication around low-carbon construction and energy efficiency significantly influenced the online narrative. More significant interactions on net-zero transition, climate tech, circular economy, mass timber housing and climate justice in 2017–2021 shaped the online climate action discourse. We find positive sentiments are more prominent and recurrent and comprise a larger share of the social media conversation. However, we also see a rise in negative sentiment by 30–40% following popular policy events like the IPCC report launches, the Paris Agreement and the EU Green Deal. With greater online engagement and information diffusion, social and environmental justice topics emerge in the online discourse. Continuing such shifts in online climate discourse is pivotal to a more just and people-centric transition in such hard-to-decarbonise sectors.

## Introduction

The Intergovernmental Panel on Climate Change (IPCC) suggests that restricting climate change to 1.5 $^{\circ }$C requires rapid and extensive changes around energy use, building design, and broader planning of cities and infrastructure^1^. The buildings and construction sector currently accounts for around 39% of global energy and process-related carbon emissions^2,3^. The International Energy Agency estimates that to achieve a net-zero carbon building stock by 2050, direct building carbon emissions must decrease by 50%, and indirect building sector emissions must also decrease 60% by 2030^4^. In a global call for net-zero strategies, a collaboration between the UN High-Level Climate Champions, the COP26 Presidency, the UK’s Department for Business, Energy and Industrial Strategy (BEIS) and the #BuildingToCOP26 Coalition announced 26 built environment climate initiatives at the Cities, Regions and Built Environment Day at the UN COP26^5^. It included net-zero carbon building commitments of over USD 1.2 trillion by the World Green Building Council^6^, and a Race to Zero through the C40 Cities Clean Construction Action Coalition including over 1049 cities and local governments, representing roughly 722 million people and committed to reducing 1.4 gigatons of CO2 equivalent by 2030^7^.

A growing body of evidence from the stakeholder community emphasises the need to incorporate public voices in global climate action to enable an equitable and just transition^8–13^. As all climate solutions will involve people one way or another, there should be a greater emphasis on socio-technical solutions and the social sciences, in addition to the continued development of complex technical solutions^14^. The European Union and the White House have also emphasised the need to create a democratised space for involving citizens at various levels of decision-making^10,11,13^. However, enabling democratic participation of people in the decarbonisation process remains a critical challenge across the local, national and regional scales^15–17^. Decarbonising the building sector is challenging as it involves a complex overlap of people, places and practices that creates a barrier to designing just emission reduction policies^18–20^.

In addition, the distinctive socio-demographic and bio-physical contexts of the built environment makes it tremendously resource intensive to use traditional survey instruments at scale^21,22^. However, the emergence of new data sources like time-series social media interaction datasets has opened up new possibilities for the large-scale cross-sectional study of such complex systems^23–25^. In this study, we use English-language social media (Twitter) data over 13 years (2009–2021, n = 256,717 tweets) to examine public reactions to climate policy events concerning building sector emissions reduction (i.e. April 2009 (before COP15) to November 2021 (after COP26)). The scale of the climate events is global and organised by intergovernmental bodies like the United Nations Framework Convention on Climate Change (UNFCCC) and IPCC.

In doing so, we look at the complex dialectic relationship between social media and climate change politics/policymaking that may shape user opinion and reactions^26^. As a result of social media, citizen journalism has increased the immediacy of breaking news; this has accelerated the speed at which politics is conducted and perceived^27,28^. With over 4.26 billion social media users worldwide^29^, the boundaries between local and global “content” has been blurred, which is increasingly seen as a critical co-production factor for climate action^30^. In this paper, we leverage this new form of digital data to capture cross-sectional variation in public sentiments and emotions following global emission reduction events concerning the building sector, thereby creating new knowledge for evaluating a people-centric and just transition with their emotional responses to climate policy processes. Our framing of “reactiveness” is based on the simultaneous consumptive and expressive characteristics of social media that make an individual’s news feeds highly personalized^25,31^. However, echo chambers exist in social media, in which individuals cluster among like-minded individuals^32,33^.

Scholars agree that while personalised news feeds contextualise relevant issues (social, political, economic, etc.), they are also a useful lens for analysing climate change opinion^25,34^. They also provide a setting to examine what sentiment users have about climate change^35,36^ and the ability to analyse how people frame their issues in the discussion^25,36^. In an ontological context, evidence shows that social media contains a greater spectrum of non-elite (general public generated content) social conversations which are not part of mainstream media organisations. However, these occur alongside elite (politicians, advocacy groups, etc.) conversations, providing a much larger intersectional bandwidth of information on public opinion which is critical for policy evaluation^25,37–39^. It is this information intersectionality that makes social media reactive to climate change content. For example, studies have shown that Twitter engagement spikes and search volume peaks around specific news stories on climate-induced weather events, high-profile media events like Al Gore’s and the IPCC’s nomination for the Noble Peace Prize^36,40^. Similarly, UNFCCC and IPCC events have shown to encourage higher hashtag use on Twitter^40^.

We conceptualise a people-centric transition in the context of the built environment as a medium of design thinking, i.e., to design interventions that are both effective in reducing embodied and operational emissions, as well as achieving wider societal goals of environmental justice: wellbeing, equity and fairness. Recent research have shown that social media platforms like Twitter can be used to derive causality discourse for users reactiveness to climate change-related events^26,41–43^. Three factors are central to understanding causality discourse on Twitter: the extreme-event factor, the media-driven science communication factor, and the digital-action factor^26^ (see SI Table 1 for detailed explanation). We operationalise this framework in this paper to evaluate three questions empirically: (i) What are the characteristics of a people-centric transition on social media towards emission reduction in the building sector over the 13-year time frame?; (ii) How has this messaging been received by users (i.e., public reactions) on social media over global climate negotiation and policy events on building emission reductions?; and (iii) How have the critical discourses changed over the temporal scale in the context of a people-centric transition?

The novelty of this paper is twofold. First, it empirically evaluates causality discourses of social media messaging of high-level policy events concerning emission reduction in the built environment. Second, it methodologically expands the use of social media interaction data from Twitter to define people-centric transition in the context of global climate action in the building sector. The methodology is a multi-method application of natural language processing (NLP), sentiment analysis and network theory (see “Methods” section). Therefore, we contribute significantly with our use of state-of-the-art computational social sciences applied to the domain of just policy design. The findings from this study will be helpful to a wide range of stakeholders who are exploring pathways for a people-centric transition and its contextualised implementation of low-carbon strategies in the context of a net-zero future.

We evaluate how different global climate action events shaped the discussions around emission reduction and low-carbon transition of the built environment using hashtag co-occurrence networks. As mentioned above, hashtags can be used to measure climate communications on Twitter^25,40^, as two overlapping processes influence the choice of using a particular hashtag: attention-seeking behaviour by users and the contagion process driven by the virality of specific hashtags^44,45^. Moreover, studies have also found that Twitter hashtags offer a strategic vantage point on social movements as they provide scalability through networked information dissemination^45^. Berglez and Al-Safaq^26^ theorise this as a causality discourse influenced by the digital-action factor, and we expand on their theory to test how certain hashtags are propagated over time. These characteristics of hashtags motivated our study to investigate how hashtag networks in the emission reduction in building discourse are shaped in the people-centric transition of Twitter communication.

## Towards people-centric climate action in building sector emissions reduction

Strategies to enable a people-centric transition in the built environment are anecdotal and not always informed by data. The proactive role of people in the decision-making process and the science-policy interface on climate change are well-studied, for example, involving recent approaches like public-private-people-partnerships (4P), value sharing governance, and information-to-empowerment approaches^30,46–51^. However, very little academic research is available on utilising public sentiments and emotions in just net-zero transitions in the building sector.

In a people-centric decarbonisation context, studies have shown that psycho-social factors like habits and attitudes are strong determinants of individual behaviour^52^. Studies like these have mentioned a habit-breaking mechanism that could help reduce emissions in the mobility sector^52^. It is also found that emotionally anchoring and objectifying climate change in media communications can enhance public engagement in the issue and form collective identities based on a mixture of emotions^53^. Furthermore, a vast pool of evidence exists on behavioural interventions for emissions reduction in buildings encompassing a range of initiatives like monetary incentives involving financial rewards to nudges and non-monetary interventions information visualisation, feedback, and social norms and motivation^54–56^. However, these studies discuss interventions as a clinical measure to reduce energy use and emissions in buildings without considering their user-emotional reaction. While capturing such emotions on a large scale can be challenging and resource-intensive, Twitter data can provide a new way of dynamically looking at how people react to climate action and policy decisions.

In addition, studies have shown that the individual-level expressions of environmental justice desires centre around the notion that people are developing a shared future. Moreover, each individual feels they have something to contribute in shaping, making and co-creating an equitable future^57^. This conception aligns with notions of personal responsibility and collective capability, extending Sen’s work on individual capabilities and collective responsibility^58^. Individualistic capabilities influence a broad range of basic needs and functions like transportation, employment, health, housing, economic opportunities, community diaspora, and political participation that shapes the collective meaning of environmental justice in the context of extreme weather impacts^59^.

Kern and Rogge^60^ further demonstrated that if political, social and psychological dimensions can support economic and technological innovations, the low-carbon transition can be achieved faster. Moreover, Martiskainen and Sovacool explore the emotions (including positive and negative feelings) associated with low-carbon energy transitions, including those in the built environment, and depict a range of reactions from joy and pride to fear and anger^61^. Similarly, Sovacool and Griffiths look at some of the negative cultural implications of building retrofits, showing how demographic aspects such as class or heritage dissuade households from pursuing energy-efficiency upgrades^62^. However, these studies approach the decarbonisation of the built environment through the transportation and mobility sector.

In a similar methodological approach, another way to study public opinion on climate policy is through social media, using data from Twitter, Facebook, YouTube, Reddit, and other social media platforms^63^. There is a growing interest in using social media to examine human behaviour, attitudes, and interactions on a real-time, large scale, within a short period, as opposed to conventional surveys or interview methods^64,65^. In addition, social media offers a unique platform where users can share their personal stories providing first-hand unmediated accounts of their lived experiences. Studies have also found that social media can lead to greater empathy with vulnerable groups and heightened response to the impacts of climate change or natural disasters while strengthening existing groups’ social ties by facilitating acts of caring, giving, and pro-social behaviour^66^. For example, a recent study explored tweets on the carbon emission trading system for multi-dimensional policy analysis in the European Union (EU). It demonstrated the importance of the public’s cognition of climate policies^67^. Furthermore, the study found that enabling public engagement (or people-centrism) in climate mitigation measures allows people to express their environmental interests, improves the transparency of policy governance and creates a space for the legitimacy of climate policies^67,68^. Kirilenko, Molodostova and Stepchenkova^42^ found that the public recognised extreme temperature anomalies and connected these anomalies to climate change through Twitter use. Similarly, Yeo, S. L et al.^43^ showed how the hashtags #globalwarming and #climatechange on Twitter influenced lay audiences’ perceptions of climate change which have important implications for climate action communication and discourse.

Also, Twitter data was used by Kim et al.^69^ to examine the public’s emotional attitudes towards nuclear energy as a low-carbon strategy. Sluban et al.^70^ used hashtag networks to explore general emotional tendencies towards ’green energy’, ’climate change’ and ’carbon emissions’. That same study concluded that more public opinion research is needed to enable a people-centric just transition. Tweets related to energy-related topics from the EU Sustainability Energy Week were used to map stakeholders’ significant energy concerns and emotional tendencies towards these issues by Bain and Chaban^71^. Veltri and Atanasova^72^ explored the network topology of climate change tweets and news media articles for automated text classification according to psychological process categories. Recently, Twitter posts that mentioned climate change in the context of three high-magnitude extreme weather events - Hurricane Irene, Hurricane Sandy and Snowstorm Jonas were used by Roxburgh et al.^73^ to derive discourses of climate denialism, criticism and polarising political ideologies. An unsolicited public opinion poll on climate change sentiments by Cody et al.^74^ used Tweets between 2008 and 2014 to explore the public emotional response to natural disasters, climate bills and oil drilling. Similarly, Debnath et al.^75^ have used Facebook posts to explore public perceptions of climate technology (in this case, electric vehicle) adoption across political, economic, social, technological, legal and environmental policy dimensions.

However, none of the above studies explores the people-centric dimensions of building emission reduction and its association with policy-led climate action. This research gap provides the primary motivation for our study. We discover five salient findings, as discussed below.

## Results

### Public sentiments in emissions reduction in buildings: Five findings

**Figure 1:**
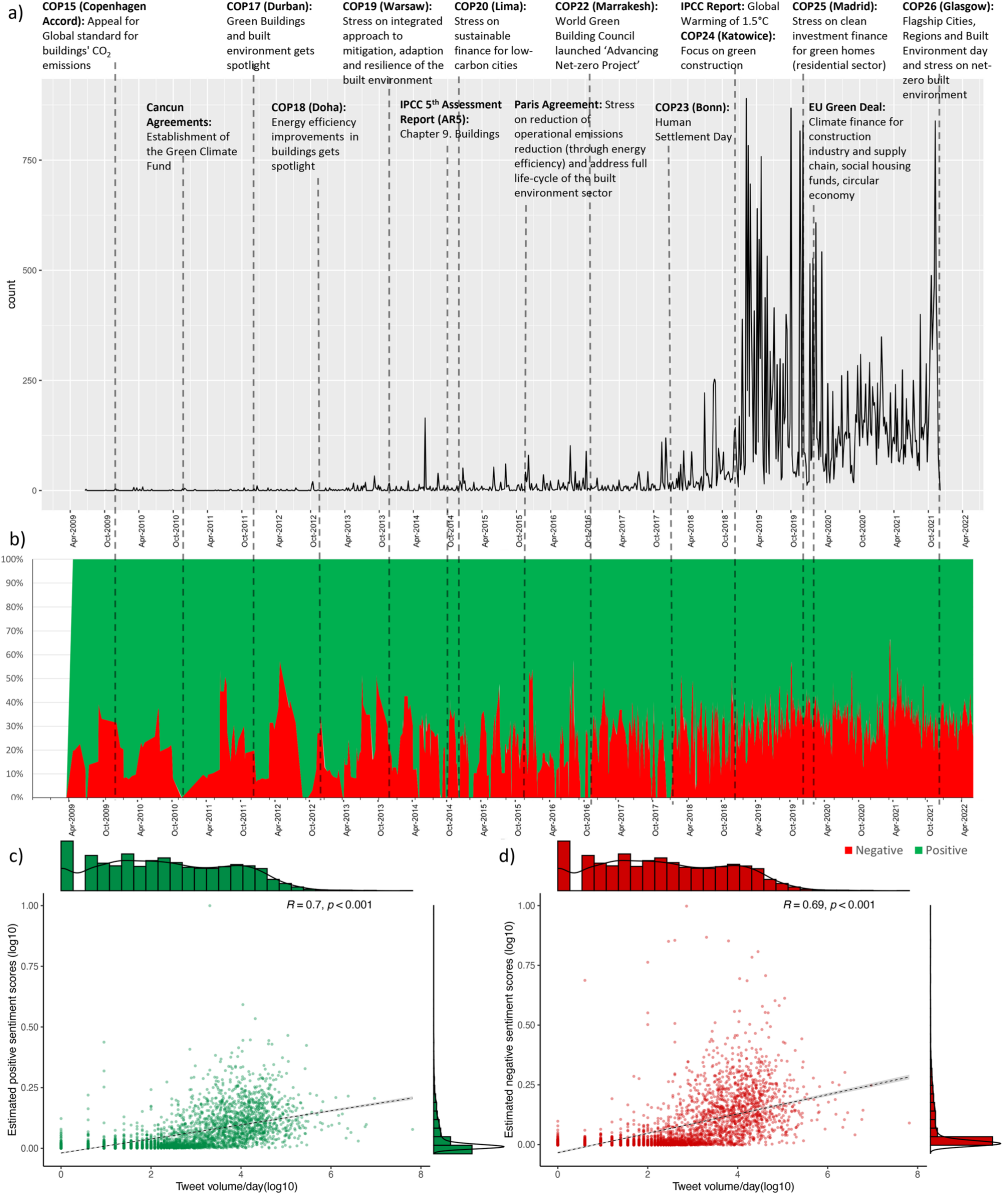
Time series of Twitter reactions concerning major climate negotiations and policy events (2009–2021). (**a**) Twitter interactions (tweets, retweets, comments) with #emission and #building; (**b**) 6-month moving average estimates of tweet sentiments; (**c**) Spearman correlation between negative sentiments with daily tweet volume. The adjusted R-squared value is 0.296, standard error is 0.192 significant at 0.001 level, (**d**) Spearman correlation between positive sentiments with daily tweet volume. The adjusted R-squared value is 0.299, and the standard error is 0.271, significant at the 0.001 level.

We begin by showing in Fig. 1 the results from the sentiment analysis of the tweets containing #emission and #building between 2009 and 2021 (n = 256,717, see "Methods" Section). Five key findings arise. First, there is a strong relationship between Twitter activity concerning the building sector and major policy events on climate change. The tweets are traced as per major climate negotiations and policy events by the UNFCCC. For example, it can be seen in Fig. 1a that there have been topics relevant to building sector emissions in the IPCC reports, but it received greater engagement following big report releases like the IPCC Special Report on Global Warming 1.5 $^{\circ }$C^76^. A similar trend in an exponential rise in Twitter engagement following a major climate communication event was also seen by Berglez and Al-Safaq^26^, which was attributed to user network creation on social media platforms. Studies have generally shown that Twitter engagement with the term ’emissions’ increases after extreme weather events^25,77^. However, our observation is uncommon for this hard-to-abate sector as we find people are also reactive to climate policy events.

Furthermore, using the causality discourse lens^26^ (which normatively relates climate communication on Twitter and its associated user engagement), enables us to infer user reactiveness through sentiment analysis following a high-level climate policy event. To use the causality discourse approach, we divided the 13-year timeline into four temporal scales (N1 (2009–2012), N2 (2013–2016), N3 (2017–*2020) and N4 (2021)) in order to evaluate Twitter causality discourses of external policy events. For N1 (2009–2013), it can be seen that the appeal for global standards for reducing building emissions began soon after COP15 in 2009. Furthermore, establishing the Green Climate Fund at the Cancun Agreement in 2010 encouraged the green building sector to spotlight the discourse around built environment-centric emission reduction at COP17 in Durban (2011). However, until COP18 in 2012, the focus was on energy efficiency and operational emission reduction benefits through an extensive focus on green buildings (see Fig. 1a).

**Figure 2:**
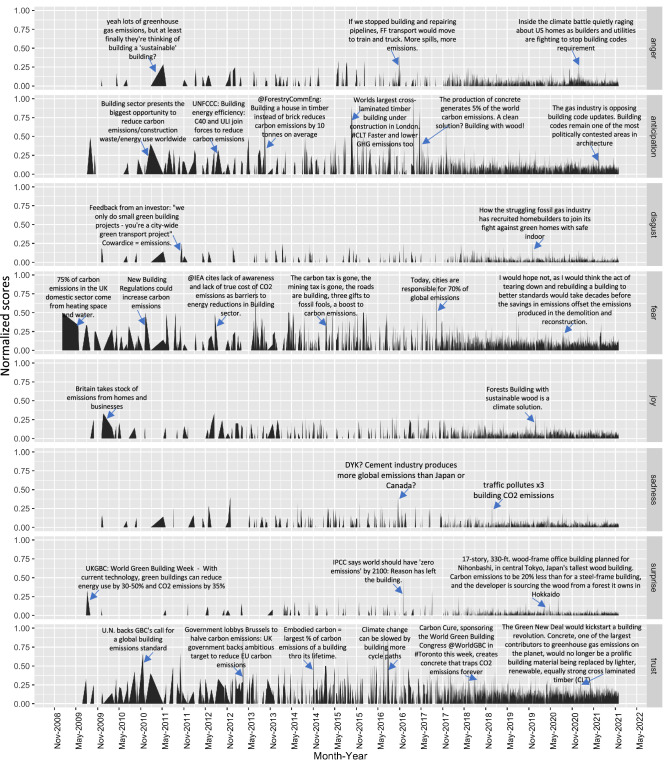
6-month moving average representation of the estimated emotion distribution in the tweets over the 13 years. Tweets corresponding to the spike in emotion score are shown across the 2009–2021 time scale. The y-axis shows the normalized score on a scale of 0.0–1.0.

Similarly, in the N2 (2013–2016) period (see SI Fig A1), policy discourse on integrated approaches to climate change mitigation, adaptation and resilience of the built environment took centre stage with the release of the IPCC Fifth Assessment Report on Climate Change (AR5) with a dedicated chapter on forecasting and long-range planning for emissions reductions from the building sector (see^78^ and Fig. 1a). This led to discussions on the need for sustainable finance for low-carbon cities in COP-20 in 2014 (see Fig. 1a). These shaped the Paris Agreement’s critical messages for the building sector: reducing operational emissions through energy efficiency and addressing the whole life cycle of the built environment sector (also mentioned in^79^), and essentially flagged wide-ranging policy discussions and stakeholder discourses on net-zero buildings and the built environment (see COP22 in Fig. 1a). Inferring from the causality discourse lens (see SI Table A1), it can be seen that both the popularity of the policy events (Paris Agreement and IPCC AR5) and its extensive media-driven science communication led to a greater engagement on Twitter.

Our results show the influence of a similar causality discourse around a higher Twitter engagement around COP-23 (N4 (2017–2020)). This COP had a specific agenda called ’Human Settlement Day’, which focused on cities, affordable housing and climate action. Such topical shifts also cause greater engagement within the users-generated network, as seen through increases in retweets, following, and followers count during N4 (shown in SI Figures A9, A10 and A11). Furthermore, with a similar causality lens, we found the tweet volume grew exponentially with the launch of the IPCC Special Report on Global Warming of 1.5 $^{\circ }$C, which stated the need to enable more profound emission reduction in the urban and infrastructure system^76^.

Moreover, the discourse on green/climate finance for residential homes got traction in COP25 in 2019, also reflected in the ’circular economy’ and ’social housing fund’ discourses of the EU Green Deal (see Fig. 1a and^80^). The growing importance of emission reduction in buildings in the global climate action and policymaking was further illustrated through the flagship ’Cities, Region and Built environment Day’ at the recent COP26 at Glasglow (2021); and its correspondingly high Twitter traffic due to heavy media coverage and user engagement driven by the digital media effect (defined as per the causality discourse lens, see SI Table A1) (see Fig. 1a).

Second, the moving-average sentiment analysis shows that positive sentiments are more prominent and recurrent and comprise a larger share of the social media conversation than negative sentiments, with few exceptions. For example, the negative sentiment share rose to almost 40% from below 10% post-COP-15. However, this share fell to nearly zero on the announcement of the Green Climate Fund (2010) (see Fig. 1b). Similarly, tweets with more than 50% negative sentiment peaked between COP-17 and COP-18 in June 2012 (see Fig. 1b). In the same period (i.e., N1: 2009–12), sentiment analysis found that tweets showed a more significant share of emotions like ’trust’ and ’anticipation’, as shown in Fig. 2 (see SI Fig A1).

The sentiment trend for N2 (2013 - 2016) also shows a higher share of positive sentiment (cumulative share of $\approx$70%), with negative peaks during the Paris Agreement ($\approx$50%, see Fig. 1b). Between January and April 2013, tweets showed emotions like high ’trust’, ’surprise’ and ’joy’, whose share fell significantly with the rise in negative emotions like ’anger’ and ’fear’ in August 2013 (see Fig. 2, SI Fig A1). The share for ’surprise’ increased during COP-19. However, the critical key emotion shared during with IPCC AR5 release was ’anticipation’ ($\approx$90%) and ’fear’ ($\approx$60%, see Fig. 2). A similar trend is seen in the tweets during the Paris Agreement, with an additional share in ’trust’ (see Fig. 2). Interestingly, the share of sadness increased after the IPCC Global Warming 1.5 $^{\circ }$C Report to $\approx$30% (N3 and N4 (2018–2021), see Fig. 2). Peaks in emotions like ’surprise’ and ’trust’ are also seen post-EU Green Deal negotiations driven by a discourse that this new deal will kick start a building regulation by replacing concrete with low-carbon materials like cross-laminated timber (see Fig. 2). Thus, the sentiment analysis showed public reactiveness in the building and emission to popular policy events, also observed by^25^.

As a general result, we see that increases in Twitter engagement (i.e. daily Tweets) have a significant correlation with both increases in negative (${R}^ 2 = 0.296$ at 99% CI) and positive sentiments (${R}^ 2 = 0.299$ at 99% CI) across the 13-year timescale (see Fig. 1c and d). Table 1 shows that the tweets with the highest negative sentiment scores contribute to spikes in Fig. 1b. Temporally an overlapping negative discourse is associated with a high carbon tax and strict building codes. At the same time, hundreds of new coal power plants are being rapidly built. These tweets also have geopolitical contexts, especially concerning China’s emissions reduction policies. More specific to the building sector, tweets from the United States showed thematic associations with stakeholder groups (like builders, utilities, and fossil fuel firms) lobbying against the implementation of climate-sensitive building codes and guidelines (see N3 in Table 1). Moreover, specific to N4 (2021), the tweets also showed the presence of climate denial and politically polarising tweets shaping an online narrative of *carbon tax as a scam*.

Third, we find a steady rise in negative sentiments by $\sim$30–40% after 2014 (see Fig. 1b). From a causality discourse perspective, this can be attributed to increased Twitter engagement with the launch of such big climate action report releases. For example, with the launch of IPCC AR5 in 2015, the share of anticipation and fear in the social media dialogue increased by $\sim$90% and $\sim$60%, respectively. Similarly, the share of sadness increased by $\sim$30% following the IPCC Special Report on Global Warming 1.5 $^{\circ }$C in November 2019 (see SI Figure A1). The trust share increased following the Paris Agreement in October 2015 ($\sim$30%) and the EU Green Deal.

Figure 2 shows that users are more reactive to specific climate action themes in the built environment. For example, a recent debate in November 2020 over lobbying of builders and utility companies over non-compliance with new building codes in the US spiked the share of “anger” in the tweets (see Fig. 2 and SI A1). In contrast, a lot more thematic diversity is seen across tweets in the “anticipation” category that spread across timber as an alternative low-carbon building material to coalition formation by non-governmental organisations to reduce building sector emissions to political polarisation over building codes implemented in the US (see Fig. 2). The tweets with the highest score for “fear” are consistent with the broader discourse of high emissions in the building sector, while the share of joy was elevated with tweets on government actions like “Britain takes stock of emissions from homes and businesses...” or “...building with wood as a climate solution...” in recent discourse. Spikes in the “surprise” score were by tweets with a socio-technical discourse that further emphasised Martiskainen and Sovacool’s^61^ findings on emotions around sustainability transition. Similarly, tweets with a high “trust” score had a common thematic focus on policy action for emission reduction, illustrating the presence of the digital action causality discourse (see SI Table A1).

Such trends provide generalised insights on the people-centric transition and reactiveness towards high-level climate policy events that shape online narratives. They show that by increasing the conversation about decarbonisation on social media, policy actions matter to the public, making them reactive through varied emotional responses (supporting the findings of^25,26,42,61^).

**Table 1:** Tweets with highest negative sentiment scores across the 13-year time scale.

Timeline	Tweets
N1 (2009–2012)	- It’s complete spin! Knocking down the old building for no reason increases the emissions associated with the project. - Errgh, tired of chatter on climate .doing something now. Building a unique emissions reduction company. Sick of hot air, fixing dirty air - cap & trade legislation “a bad fit for addressing the building human influence on the climate,” because of foreign emissions. - Typical stupid politicians!! lower Aussie CO2 emissions by(wait for it) building new coal plants! - How the heck? You are building more coal-fired power stations! Less emissions, yeah right Guvvanunt!!!! - No emissions and no grid connection required - think this is the future of buildings? No
N2 (2013–2016)	- Emissions building faster than we thought’ they won’t rise enough. China’s building 500 coal stations over 10yrs. Can’t you read? emissions risen despite the “action”. - And building new #pipelines is not how we get there: CO2 emisions must be 0 by 2070 to prevent #climate disaster - Now that his own State Dep’t has told him after 2 exhaustive reviews that NOT building Keystone will increase emissions, Obama says - The UK is building the worlds first power plant that might have *negative* emissions - Bigger problem w/ carbon capture (CCS). IEA estimates say it will be 20% of emissions cuts...but nobody is building CCS at scale. - Typical Wolfsburg performance. Building up the hype but never really living up to it. More fraudulent than VW’s CO2 emissions report.
N3 (2017–2020)	- It’s time we say NO to Trudeau’s Carbon Tax in Canada. Time everyone started a protest movement like in France. - Not only is no one cutting CO2 emissions, the world is building 1200 new coal plants and producing record amounts of oil - Problems with nuclear power include risks in mining and transportation, nuclear weapons, the cost of building a power station, - Foreign Ministers should announce that we are in a state of emergency, a climate emergency, - and that’s if global warming is real We cannot address #ClimateChange w/o phasing out gas appliances in buildings. Period. - Inside the climate battle quietly raging about US homes. The gas industry is opposing building code updates - Any mentions of China building 300 coal power plants over the next 30 years? And their Paris “commitment” not having them decreasing emissions for over a decade?
N4 (2020–2021)	- China will keep building coal plants while Biden raises energy costs for Americans by crushing fossil fuels. - Invest in carbon capture tech. It looks ridiculous to pledge cutting carbon emissions while building pipelines and fracking. - ITS A SCAM by Liberals....You are thieves and fraudsters. Carbon Scam Tax wont and hasnt made any difference at all in global GHG emissions. - Please cancel the dirty-fueled Peabody Peaker. You can’t be building a new fossil-fuel powered electric plant and meet emissions reductions goals. #StopPeabodyPeaker - CLIMATE CHANGE IS NATURAL! We only make 5% of CO2. We produce GIGA-TONS of AEROSOLS, offsetting our CO2 emissions. - Stop destroying American industry over this FALSE threat. Solar cells are a net energy DRAIN when you count ALL costs. - “Liberals have pitched themselves as climate leaders with policies like the carbon tax and setting a target of net-zero by 2050. But they have presided over six years of growing greenhouse gas emissions, and they are building a new bitumen pipeline...”

**Figure 3:**
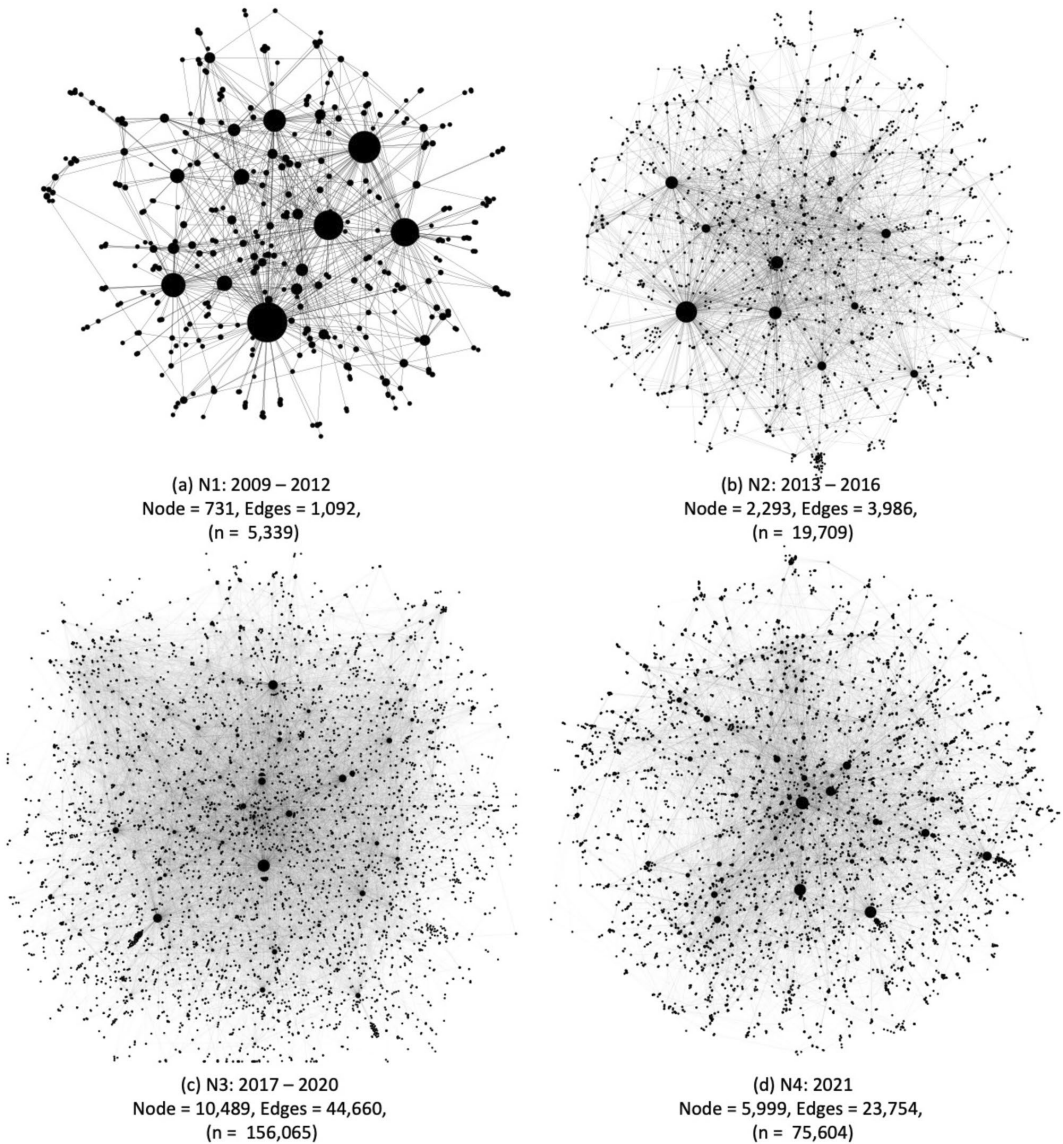
Topologies of hashtag co-occurrence networks (N1–N4) across the 13-year timeline (2009–2021) denoting diffusion of climate policy events into the public domain. The size of the node is respective to its frequency in a particular network.

Fourth, an exponential increase in the number of edges and nodes of the resultant temporal network demonstrated the topical diffusion of high-level climate policy discussions in the public domain (see Fig. 3, network characteristics in SI Table A2). Building on network theories of information diffusion^81,82^, here we refer to diffusion as an increased number of inter-related hashtagged topics in the tweet network containing #emission and #building. Moreover, we constructed these networks as co-occurrence networks that fundamentally implied that user interactions drive hashtag expansions. Thus, emphasising that information diffusion occurs through the growth of co-occurring hashtags with the base hashtags (emission and building).

**Figure 4:**
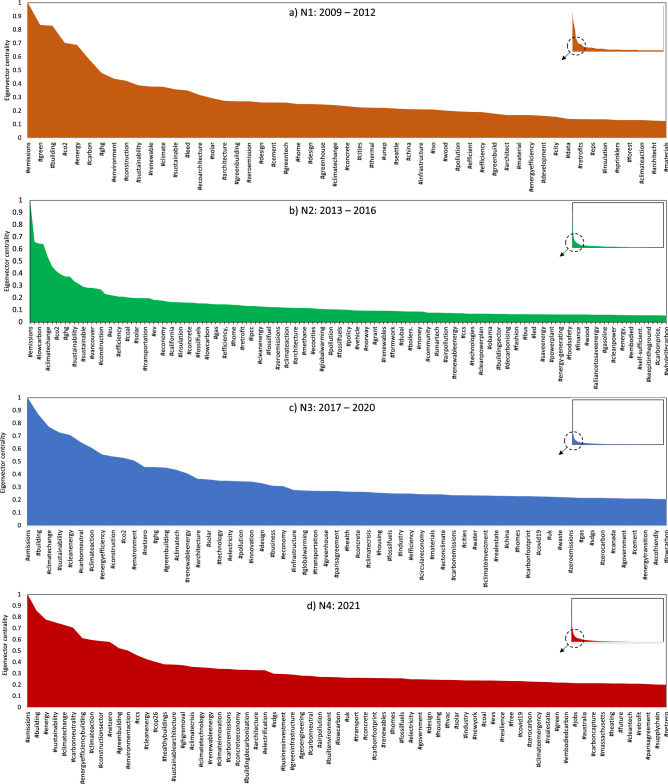
Eigenvector centrality score ( $$> 0.01 $$) distribution with nodes (hashtags) in the N1–N4 network. A score of 1 indicates highest influence of a specific hashtag, while 0 implies hashtags of least influence. This measure demonstrates changing online public discourse through hashtags over the 13 year of climate policy action. We see mid-end of centrality scores (0.1– 0.3) to be most dynamic across the networks and use it as a basis for analysis in this paper. Extended results are presented in SI Figures A2, A3 and A4.

The nodes represent the changing ego centres of the specific hashtags that shape public discourse on carbon reduction in the built environment. For example, in Fig. 4a, the larger nodes for N1 show the ego centres for hashtags #emissions, #carbon, #building and #environment, which denote the trending social media discourse for 2009–2012. This network expands in N2 (see Fig. 3b and SI Figure A2) with trending hashtags such as #climatechange, #building, #energyefficiency, #ghg, #transportation, #greenbuilding and #actonclimate, denoting the growing public interest for climate action in the built environment between 2013 and 2016. This network expansion indicates increased public awareness and engagement toward climate change and carbon reduction in the built environment, which can be seen significantly expanding in Fig. 3 (N3 and N4).

We also find that the network modularity increases over time (i.e., 0.444 for N1 to 0.674 for N4), indicating more connections between the nodes within modules but sparse connections between nodes in different modules. It implies that with expanding network (N1–N4), hashtags co-occurrences occur through greater intra-connections - indicating user engagement over similar topics in groups. Therefore, we see specific hashtags become more central to the Twitter conversation over time, which is further explained through the use of eigenvector centrality scores.

**Figure 5:**
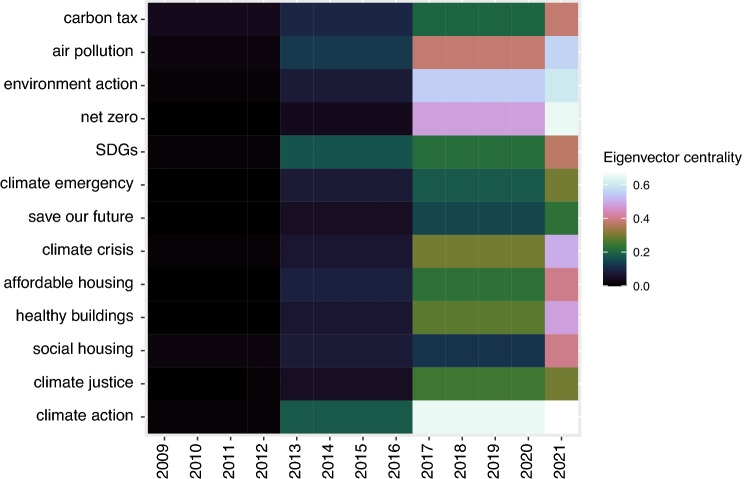
An eigenvector centrality heatmap showing temporal shifts in the online discourse through common social and environmental justice hashtags (#, y-axis) across the 13-year period (2009–2021).

N3 (see Fig. 4c and SI Figure A3) further shows the diffusion of the above hashtags into more specific climate action topics like #climatetech, #netzero, #renewableenergy, #innovation, #health and #climatecrisis, documenting that higher-level policy action translates into more specific climate communication toolkits that create action-oriented online communities, for 2017 - 2020. This expansion of online climate communities is also verified through higher modularity scores for N3 than N2 (see SI Table A2). The N4 was specific to the recent UN COP-26 in 2021, after which new hashtags like #buildingstocop26, #woodforgood, #housingcrisis and #climatejusticenow appeared (see SI Figure A6), showing the growing public interest in carbon reduction as a route to enabling social and environmental justice. Common environmental justice hashtags across the 13 years with their corresponding eigenvector centrality score are illustrated in Fig. 5.

Fifth, tweets’ core topics change over time as new innovations, technologies, or issues emerge. We specifically see this topic dynamism for mid-range centrality scores (0.1–0.3), and the hashtags in the higher score range do not change that much across N1 to N4 (see Fig. 4). For example, in the mid-end of the eigenvector centrality score (0.1–0.3), additions like #carboncapture and #masstimber were new in N4. They provided a critical clue toward the changing focus on the life cycle of emissions and sequestration through carbon capture and storage (CCS), natural materials, nature-based solutions and mass timber housing (see Fig. 4d and SI Figure A4). However, as these new emergent hashtags do not become highly central over time, it implies that the core online discourse of decarbonising the building sector does not shift significantly with the conversation around new technological or process-related innovations.

There are significant overlaps between N3 and N4 regarding hashtags with high eigenvector centrality values as they are part of current climate policy and emission reduction conversations in the building sector (see Fig. 1a). New hashtags in N4 can be tracked in Fig. 4d. This figure includes #ccs and #climatetechnology in the higher score range (0.6–0.3), a shift from N3, also demonstrated through network expansion in N4. This shift can be attributed to its association with #cop26, which increased its network influence. Additions also include #businessinnovation, #geoengineering, #concreteeconomy in the same range. Moreover, it can be seen from Fig. 4d that greater emphasis on #homes, #retrofit, #supplychain in the mid-score range (0.1–0.3) indicates a shift in the online discourse towards residential sector emission reduction efforts (see Fig. 4d), which is a critical demand-side decarbonisation topic.

Also, some of hashtags associated with #cop26 in the N4 network are #buildingtocop26, #woodforgood, #healthyclimate, #housingcrisis, #scaleupnow, and #climatejusticenow (see Fig. 4d and SI Figure A6). It indicates a paradigm shift in the emission and building policy narratives towards broader social and environmental justice contexts. For example, N3 and N4 #masstimber and #woodforgood featured relatively higher eigenvector centrality values showing emerging themes in the building emission reduction discourse: alternate low-carbon materials to concrete construction. Similarly, the housing crisis, healthy climate, scale-up, and climate justice are related to the social justice movement associated with global affordable and healthy social housing narratives.

We further show this thematic evolution to social and environmental justice themes in Fig. 5. For example, we found an increase in eigenvector centrality scores for hashtags like affordable housing, save our future, climate justice, and carbon tax from 2017. More specifically, an increase in the centrality scores is seen for #climateaction, #climatecrisis, #healthybuildings and #netzero across N1 to N4 period, demonstrating diffusion of such topics in users’ Twitter network. This provides crucial empirical evidence demonstrating that the online climate action discourse has become broader and people-centric over time.

## Discussion

We have performed a data-driven analysis of the reactiveness of social media users to global climate negotiation and policy events on emissions reduction efforts in the building sector. The analysis used 256,717 tweets mentioning #emission and #building over a 13-year time frame (2009–2021). Our results show that social media users are reactive to high-level policy events by UNFCCC in the building sector. We found five results characterising how the people-centric transition in the built environment is influenced by social media use. As a general trend, we find that increases in Twitter engagement (i.e. daily Tweets) have a significant correlation with increases in both negative (${R}^ 2 = 0.296$ at 99% CI) and positive sentiments (${R}^ 2 = 0.299$ at 99% CI) around building sector climate action (see Fig. 1). We find positive sentiments grow over time as Twitter engagement increases exponentially post-2014. We also observed a rise in negative sentiments by 30–40% since 2014, with spikes caused by tweets on environmental injustice (like the world continues to build coal power plants and oil pipelines), non-compliance by influential actors (like governments, fossil fuel industry, etc.) and climate denialism (see Fig. 2). We also see a general trend where emotions like anger, fear and sadness following high-visibility policy events like the Paris Agreement, IPCC Reports and EU Green Deal announcements - potentially caused by the digital action factor and the media-driven science communication factor as theorised by Berglez and Al-Safaq^26^.

Mapping tweets that caused a spike in the dynamic emotional response revealed that public concerns triangulated around inaction towards emission reduction, fairness of carbon tax, the politicisation of building codes (distinctively seen for the US) and concerns of environmental degradation (see Fig. 2). This triangulation demonstrated a strong environmental justice discourse amongst the Twitter users (supporting the findings of^62,83^). On the other hand, the tweets corresponding to higher anticipation scores showed a strong discourse on innovative emissions reduction strategies uncommon in the building and construction sector-for example, enabling construction with alternate building materials like cross-laminated timber (CLT), implementing climate-sensitive building codes and circular economy. We specifically see these terms emerge and create a broader tweet network in N3 and N4 (see Fig. 3 and SI Figures A5 and A6)). Moreover, in these hashtag co-occurrence networks, we further see the emergence of broader interconnected low-carbon transition and environmental justice terms like social housing, climate justice, wood for good, save our future, climate emergency, climate tech, affordable housing, healthy buildings, etc. (see Fig. 4 in N3 and N4) which were either absent or in extremely low salience in N1 and N2 networks. We further showed these temporal shifts in environmental justice-thematic hashtags in Fig. 5.

Such network expansion due to increased Twitter engagement and subsequent information diffusion in the building sector emission reduction supports existing research on social network theories emphasising such diffusion is critical for social integration^81,82^. Our results demonstrating the emergence of environmental justice themes in N3 and N4 (see Figs. 4 and 5) further emphasise that greater engagement can influence communication on emissions reduction. Our research can inform future emission reduction strategies in the building sector.

Thus, our findings provide two vital empirical proofs in support of increasing climate communication and engagement in hard-to-decarbonise sectors. First, greater digital engagement shapes public perceptions of emission reduction as demonstrated through expanding hashtag network concerning #emission and #building (see Figs. 1 and 3), enabling people to be at the centre of such emission reduction efforts. Second, people are concerned about climate action in this sector as their engagement drives the narratives around justice and fairness, or at least we see users are engaging with relevant hashtags, as seen through emerging terms from energy efficiency and green building in N1 and N2 to social and environmental justice concerns in N3 and N4 (see Figs. 3, 4 and 5). The COP26 was extraordinary as the N4 hashtags attributed to the intersection of public health, circular economy, affordable housing, and decarbonisation of the built environment (see SI Figure A6). Although these mid-eigenvector centrality scores (0.1–0.3) capture these topics as emerging themes, it supports our assumption that in a people-centric transition of the built environment, the public is concerned with both emission reduction and achieving goals of environmental justice.

However, reflecting on the current Twitter user base ($\approx$211 million users globally), we found approximately a quarter-million or so tweets on emissions in the building sector during our analysis period, indicating that these issues are still low salience. So, one crucial task for policymakers is to immediately raise the salience of these issues and develop communications strategies to emphasise the importance of climate action in hard-to-decarbonise sectors like the building sector.

## Conclusion

Building sector decarbonisation is challenging, and it is an urgent global call for the scientific and policy community. Our study shows that greater social media engagement can steer the online discourse on emissions reduction in this sector from demand-side techno-solutionism to focus on environmental justice while expanding the climate action network. This finding has critical implications for designing people-centric policies that enable the public to be at the heart of the emission reduction discussions^14^. We show that people are reactive to high-level climate actions on social media, as measured through the dynamic sentiment analysis over 13 years and the expansion of user communication networks. This salient finding implies that policy actions are no longer isolated events in this digital age and demand two-way communication.

At a broader level, this paper showed people are reactive to high-level climate action in a hard-to-abate sector and should be included systematically in emission reduction decision-making, supporting current debates on the democratisation of climate action. Failure to include public voices in this policy debate may lead to environmental and social injustices. Methodologically, we also show that social media provides a new type of cross-sectional public datasets for climate policy evaluations and can be easily reproduced in other sectors to support existing econometric methodologies.

We would like to note here that our Twitter dataset is generalisable to a specific population of users tweeting about these topics for which these issues matter. Thus, our conclusions hold true for this user base who engage with Twitter communication containing #emission(s) and #building(s). We studied the opinion of those for whom the topic was salient. It was beyond this study’s scope to examine the topic’s relative importance for those who do not engage with the above hashtags. Thus, critical conclusions from this paper are: emissions reduction decision-making for hard-to-decarbonise sectors need not be isolated; people are reactive to high-level climate action events on social media platforms. People engage and expand their network on these platforms to express matters vital to them. In our case, it was the context of environmental and social justice with climate action in the building sector. Thus, digital tools like social media can be critical for enabling a people-centric climate action.

This paper notably has some limitations. For example, we relied on Twitter’s publicly available dataset through v2API, which can have embedded socio-demographic representation bias. To reduce such biases, we used a restricted hashtag query design using ’AND’ operator to capture the cross-sectional breadth of the issue in the buildings sector, per the best practice recommendations^84–86^. We used the English-language-based NRC lexicon for sentiment analysis, which is well-recognised in the literature. However, it is limited to its current lexicon database. It does not account for complex human emotions like irony or sarcasm. We cross-validated at least 40% of the Tweets with human readers for sentiment matching. It remains a future extension of this study to employ survey methods and participatory workshops with different stakeholders to capture the rich narrative of grounded emission reduction efforts following such high-level policy events. Furthermore, research is necessary to compare the impact of on-Twitter versus off-Twitter activities on policy reactions and develop a methodology of cross-validating online and offline discourses with greater accuracy. Finally, we expand this niche reactive public policy analysis field using computational social sciences.

Our study fills a critical research gap using a novel data stream. Moreover, it paves the way for future research on people-centric climate action across hard-to-decarbonise sectors. Finally, this paper emphasises explicitly that social media platforms can help amplify such efforts by engaging and improving the public understanding of the climate crisis.

## Methods

Using a unique Twitter dataset, this study uses a data-driven mixed-method methodological approach to understanding public reactiveness to high-level climate policy events by UNFCCC concerning emission reduction in the building sector. The mixed-method approach consists of two critical stages. First stage is the empirical setup of a theoretical background based on existing social media climate communication literature^25–27,36^, especially expanding on a critical discourse theoretic lens^26^ and applying it to our climate policy reactiveness evaluation. The second stage is the quantitative treatment of 256,717 tweets over 13 years (2009–2021) using natural language processing (NLP), sentiment analysis and hashtag extraction, and network topology analysis based on eigenvector centrality and hashtag co-occurrence mapping. These stages are explained in detail below with supporting information presented in SI Section 2.

### Data source

Digital social media platforms like Twitter have gained extensive public popularity among researchers as an effective source of data on public opinion about environmental issues and climate policies as it enables a diverse range of user-generated content (like texts, images, pictures, audio, video and live conversation)^63^. In addition, tweets offer advantages over traditional methods (including online and face-to-face surveys) for exploring public perception and attitudes^65^. For example, Twitter users can independently publish and deliver UGCs of their choice, adding depth to their opinions^87^. Moreover, users can interact through conversational replies, retweets, and likes, showing the relationships and reflecting the social nature of information transmission^87^. Furthermore, as a data platform, Twitter can gather real-time data on an extraordinary scale and dimension (i.e., time, location, user attributes) and echo public awareness and response to social and environmental policies, facilitating discussions and information propagation^88^. Finally, Twitter allows academic researchers to collect and analyse data from their platform.

In this study, we use Twitter’s latest v2API^89^ to collect historical tweets for 13 years. We used the R-programming language to build the query parameters for data collection using the academictwitteR v0.3.0 package. The tweets are downloaded as separate JSON files for a tweet- and user-level information separately on each query. These data packets are then bound into an R data.frame object or tibble for further analysis in the R environment. Due to the development and expansion of Twitter between 2009 and 2021, the number of Twitter users has increased significantly. We normalise this effect using ratios rather than absolute figures across the time scale. Detailed sample characteristics are illustrated in SI Section 2 (Figures A7 to A11).

As a search query, we used two specific hashtags (#) ’#emission’ and ’#building’ (and its plural forms) with the logical operator ’AND’ to capture any available English-language tweets in public Twitter v2API domain during this 13-year timeframe without any geographical restrictions. This produced a dataset of 256,717 tweets and retweets containing the above hashtags from 188,096 unique user accounts with an exponentially increasing trend from 2009 - 2021. We kept the search query restricted by design, as more general queries would expand the size of the datasets greatly, leading to downstream problems like noise and computational/analytic difficulties. Similarly, more complex queries (i.e. nesting) would lead to complex datasets, as well as shifting the focus from the building sector. Our query generates a traceable dataset, that likely has less noise than a a much larger dataset would if it were generated by less restrictive query, and which allows for straightforward analysis of the data. We specifically use hashtags as they are a critical communication tool on Twitter and have become an essential part of Twitter-led data analysis^85^. Users deploy hashtags to annotate the content they produce, allowing other users to discover their tweets and enable interaction on the platform^90–94^. Also, adding a hashtag to a tweet corresponds to joining a network or community of users (Tweeters) discussing the same topic. Finally, hashtags are also used by Twitter to calculate trending topics, which encourages the users to post and engage in these communities^91^. By tweeting a hashtag, users explicitly annotate their tweets for a specific network of Tweeters, or communities^92,94^.

### Natural Language Processing

The tweets were processed with an NLP workflow using the tidyverse v1.3.1 and tidytext v0.3.2 packages in R. The workflow consisted of text pre-processing, feature extraction for n-grams and sentiment analysis. The pre-processing stage consisted of tokenisation, stemming and lemmatisation. In NLP, tokenisation refers to breaking down the given text into smaller units in a sentence called token^95,96^. Stemming in NLP is a morphological technique that breaks words into their root form^96^. Finally, lemmatisation is another normalisation technique used to reduce inflectional forms of words to a common base form^96^. It differs from stemming as it uses lexical knowledge bases to get the correct base forms of words^96^. At this NLP pre-processing stage, we removed the stopwords using the tm v0.7-8 package in R. Stopwords are the most common words in any language (like articles, prepositions, pronouns, conjunctions, etc.) which do not add much information to the text. For example, common stopwords in English are “the”, “a”, “an”, “so”, and “what”^96^. This workflow extracted the cleaned base form of words from the 256,717 tweets. In addition, it generated a document-term-matrix (dtm) needed for sentiment analysis.

Parallel to this pre-processing, we isolated individual hashtags from the tweet data corpus. The hashtags were extracted from each tweet using a feature extraction-like data pipeline where the tidygramr v0.1.0 package prepares n-gram models. We extracted hashtag unigrams from each tweet (n = 13,743) and stored them as a separate dtm that included feature vectors of #ngram and the number of times it is repeated in an individual tweet (called ‘freq’). Both ‘#ngram’and ‘freq’ were later used to create the hashtag network graphs. During this unigram feature selection process, we also excluded ‘#emission’ and ‘#building’ to reduce over-representation biases in the dtm.

We used the NRC Word-Emotion Association Lexicon^97^ for the sentiment analysis of the tweets through the syuzhet v1.0.6 package^98,99^. It consists of a list of English words and their connotations with eight basic emotions (anger, fear, anticipation, trust, surprise, sadness, joy, and disgust) and two sentiments (negative and positive); the list of corresponding words/terms to the specific sentiment and emotions can be found here:^98^. The derived sentiment scores were then scaled between 0 and 1 (feature scaled) through a min-max normalisation function (see eq. 1) in R to visualise its strengths across the tweet time series. In addition, the time-series of sentiments were represented through moving average decomposition of 6-months trends (see eq 2).

1 $$\begin{aligned} normalized.values= & {} \frac{value - minimum}{maximum - minimum} \end{aligned}$$

2 $$\begin{aligned} T_t= & {} \frac{1}{m}\sum _{j=-k}^{k} \end{aligned}$$

where $$ m=2k+1$$. The estimate of the trend-cycle at time *t* is obtained by averaging values of the time series within *k* periods of *t*. All of the code necessary to reproduce the pre-processing and analysis are available at https://github.com/Ramit1201/EmissionReduction.git.

### Network topology analysis

In the previous step, we extracted 13,743 unique hashtags n-grams from theQ2 256,717 tweet corpus. These #ngrams provide the basis for constructing hashtag co-occurrence networks for #emission(s) and #building(s). The hashtag co-occurrence networks were produced for four distinct timescales, N1 (2009–2012), N2 (2013–2016), N3 (2017–2020) and N4 (2021). Co-occurrence networks are a graphical representation of how frequently variables appear together^100^. In our hashtag co-occurrence network construction, we measure how frequently (‘freq’) specific hashtags (as #ngram) are presented in a single tweet. A node represents each hashtag in the network, and the co-occurrence between two nodes represents an edge-weighted by its frequency. Key steps involved in the construction of the co-occurrence networks are building a weighted edge list, conversion to an undirected network containing #ngrams connected by edges indicating when these #ngrams were listed together and then visualising the network using Gephi v0.9.2. Weighted degree values for each node measure the importance of an n-gram. A higher value indicates a greater influence of that n-gram (denoted through larger labels) in that network. The undirected network topologies were evaluated based on metrics commonly used in social network analysis research^101^.

Modularity is at the core of the most popular algorithms for community detection. It measures the structure of a graph *G* where each partition of the vertices has a modularity score. With higher scores indicating that the partition better captures community structure in G^102,103^. Modularity as a metric *Q* can be expressed as (see eq. 3)^104^,

3 $$\begin{aligned} \user2{Q} = \sum _{i}\left( e_{jj}-a_i^2\right) \end{aligned}$$

where $\ e_{jj}$ is the fraction of edges in the network that connect vertices in partition *i* to those in partition *j*, and $a_{ij} = \sum _ie_{jj}$^104^.

Eigenvector centrality measures the influence of a node in a network. It evaluates a node’s importance while considering the importance of its neighbours^105^. It is based on the principle that links from important nodes are valued more than links from trivial nodes. All nodes start equal; however, nodes with more edges start gaining importance as the computation progresses. Their importance propagates out to the nodes to which they are connected. Through iterative computing, the values stabilize, resulting in the final values for eigenvector centrality^106^.

The clustering coefficient is defined in graph theory as a measure of the degree to which nodes in a graph tend to cluster together^107^. For an undirected graph, the global clustering coefficient *C* is estimated in terms of the adjacency matrix *A* (see eq. 4),

4 $$\begin{aligned} C = \frac{\sum _{i,j.k}A_{ij}A_{jk}A_{ki}}{\sum _{i}k_i\left( k_i-1\right) } \end{aligned}$$

where $\ k_i = \sum _{j}A_{ij}$, is the number of neighbours of a vertex, *i* and *j* are vertices of the graph.

Degree centrality measures the number of edges connected to a node, which is a widely used centrality measure. It is expressed as an integer or count and assigns an importance score based simply on the number of edges held by each node. The nodes with a higher degree are central^106^. Mathematically it is represented in eq. 5,

5 $$\begin{aligned} D(i) = \sum _{j}m(i,j) \end{aligned}$$

where $\ m(i,j)=1$, if there is a link from node *i* to node *j*. Graph density measures how many ties between parameters exist compared to how many ties between parameters are possible. The density of an unidirected graph is presented in eq. 6,

6 $$\begin{aligned} \text{ Unidirected } \text{ Network } \text{ Density } = \frac{{\text{ Total } \text{ Edges }}}{{\text{ Total } \text{ Possible } \text{ Edges }}} = \frac{{\text{ Cardinality }}}{{\text{ Size }}} = \frac{m}{n(n-1)/2} \end{aligned}$$

where *n* is the number of nodes in the network.

The networks were optimised using the ForceAtlas2 (FA2) algorithm based on a force-directed layout that simulates a physical system to spatialise a network^108^. Nodes repulse each other like charged particles. At the same time, edges attract their nodes like magnets. These forces create a movement that converges to a balanced state with higher connected nodes placed centrally while nodes with lower connectivity are placed towards the network’s periphery^108^. This final (optimised) network configuration is expected to help interpret the data. The refinement of the network visualisation was performed by using the *linlog*, gravity and overlapping prevention layout settings in Gephi for the FA2 algorithm [a detailed mathematical background for FA2 is provided by Jacomy et al.^108^. Additionally, further visual refinement of the networks was performed using functions like Noverlap, and labeladjust layouts^109^.

### Ethics approval

This research was reviewed by the Institutional Review Board at the Judge Business School, University of Cambridge (20-064) and at the California Institute of Technology (21-1169). Twitter was informed about this research during the v2API request.

## Supplementary Information

Supplementary Information.

## Data Availability

The datasets analysed during the current study are available in the Open Science Framework repository https://doi.org/10.17605/OSF.IO/QC453. The user identifiers are anonymized as per Twitter’s developers policy and GDPR rules https://gdpr.twitter.com/see here.
