# Supplementary material for: Social media enables people-centric climate action in the hard-to-decarbonise building sector
Source: Sci Rep. 2022 Nov 17;12:19017. doi: 10.1038/s41598-022-23624-9 (PMC9671910; doi:10.1038/s41598-022-23624-9)
Supplement: Supplementary file 1 — Supplementary Information. [file 41598_2022_23624_MOESM1_ESM.pdf]

Supplementary Information (SI)

Social media enables people-centric climate action in the hard-to-decarbonise building sector

Ramit Debnath, Ronita Bardhan, Darshil U. Shah, Kamiar Mohaddes, Michael H. Ramage, R. Michael Alvarez, Benjamin K. Sovacool

Section 1. Results

Table A1 shows the theoretical framework on Twitter causality discourse that informs our time-series interpretation of high-level policy events and Twitter engagement. We used this framework to qualitatively support our data-driven analysis and improve the generalisability of the findings.

Table A1 Causality discourses of climate communication on Twitter, as per Bergez & Al-Safaq (2020)

Extreme-event factor:	the mediated highlighting of extreme weather, in a general sense or with a focus on previous or ongoing events.
Media-driven science communication:	media information mentioning 'causality discourse' deriving from parallel media channels/platforms, be they traditional news channels, newspapers, or broadcast-radio news, but also political campaigns, blogs, think tanks, organisations, etc.
Digital-action factor:	individual users' networked generation of 'causality discourse.'

The table A2 presents key network characteristics of N1 to N4 that supports the network theory-driven findings in the manuscript. It also provides extended data for Figure 3 in the main manuscript.

Table A2 Macro-network characteristics

Network	Year	Density	Avg. degree	Modularity	Avg. clustering coefficient
N1	2009 – 2012	0.015	2.988	0.444	0.825
N2	2013 – 2016	0.005	3.447	0.479	0.784
N3	2017 – 2020	0.002	8.516	0.667	0.838
N4	2021	0.002	7.919	0.674	0.846

Fig A1 illustrates a time-series 6-month moving averages of embedded emotions in the tweets as a function of the public reactiveness to high-level climate policy events. It's extended version is presented in Figure 2 in the main manuscript with corresponding tweets having highest emotional scores. Fig A1 shows emotions as a ratio of emotional shifts in the public reactions.

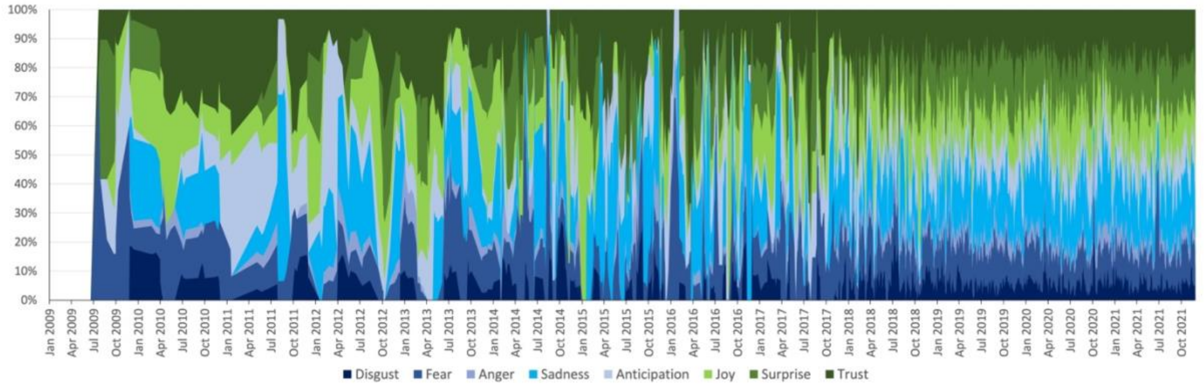

Fig A1: 6-month moving average share of emotions across the 13-year time scale.

Figures A2, A3 and A4 qualitatively shows the strength of hashtags in its specific network (N1 and N2). The size of the word corresponds to its centrality scores in the network, i.e., the larger the size of the word the greater is its influence in the online discourse. We stress on the mid-range eigenvector scores as they denotes the most dynamic hashtags in our data corpus over the 13-year period, while the hashtags with high centrality scores subjectively remain same through the N1 to N4 network.

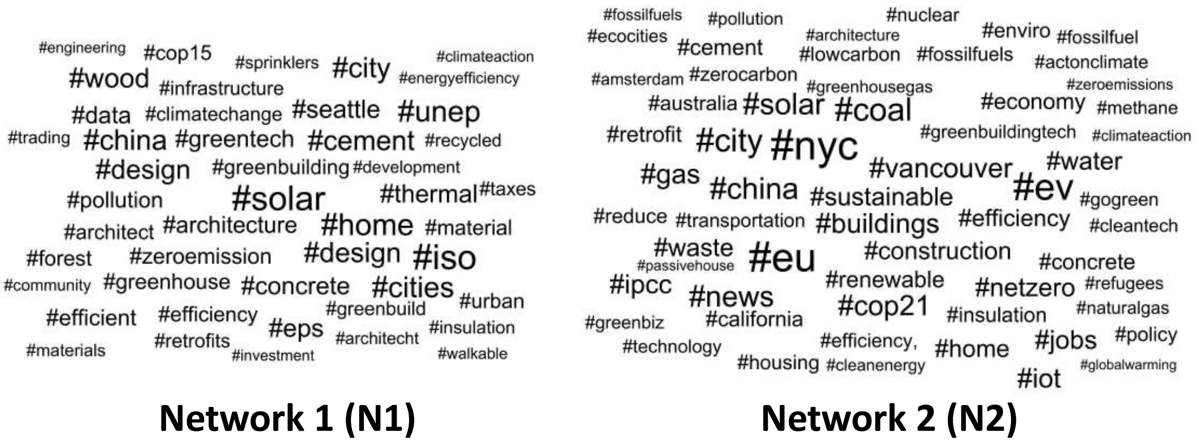

Fig A2: Hashtags for N1 and N2 with eigenvector centrality scores in the range of 0.1 to 0.3.

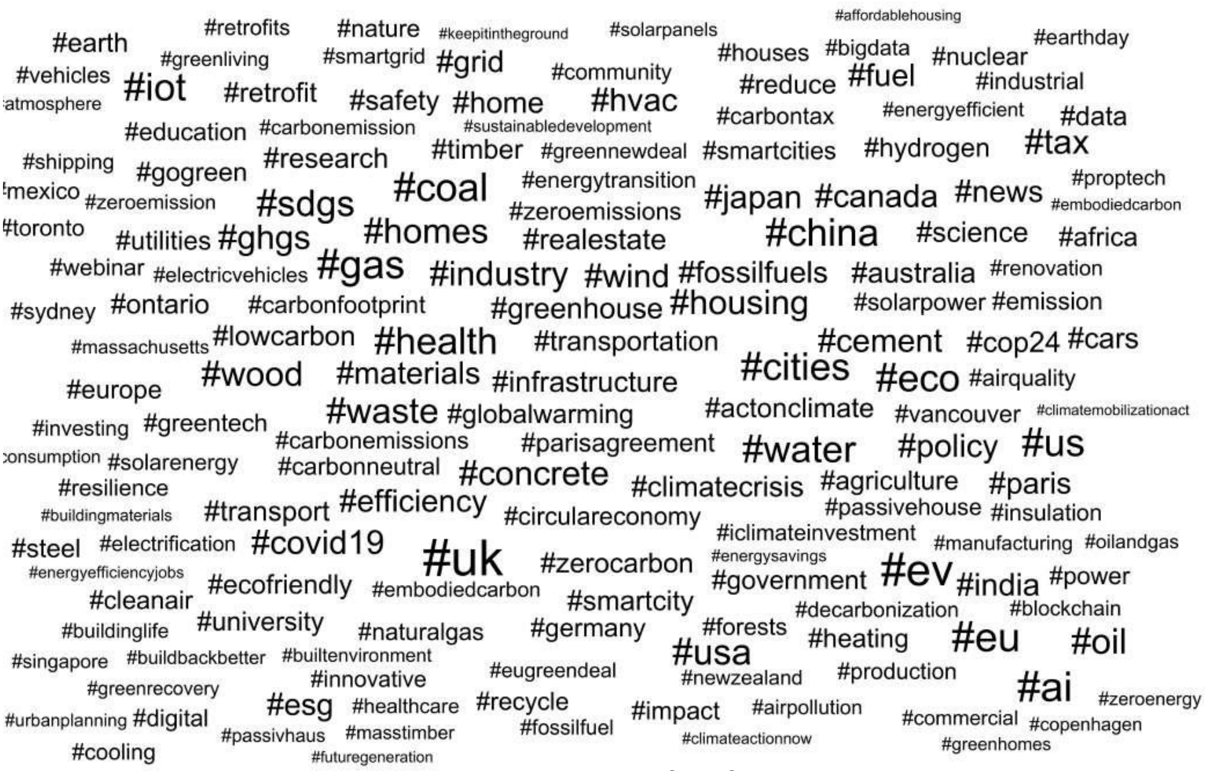

Network 3 (N3)

Fig A3: Hashtags for N3 with eigenvector centrality scores in the range of 0.1 to 0.3.

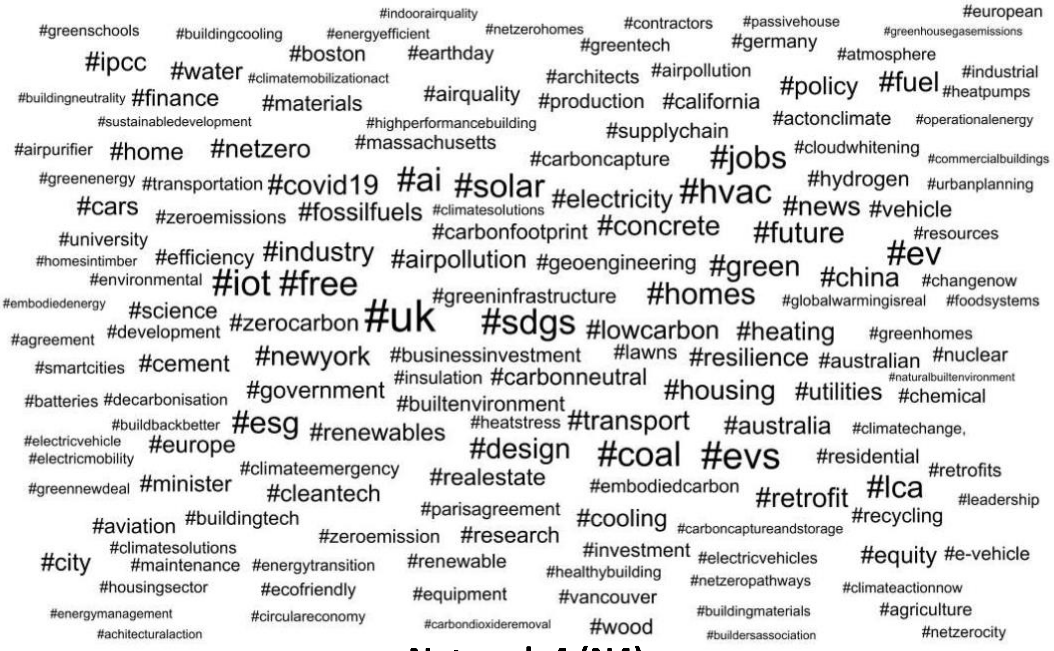

Network 4 (N4)

Fig A4: Hashtags for N4 with eigenvector centrality scores in the range of 0.1 to 0.3.

Fig A5 shows the complex network topology for N3 (2017 - 2020). We see distinct hashtag clusters on climate action, energy efficiency and emission in buildings. These clusters were absent in N1 and N2 and provide evidence of an information diffusion effect due to greater Twitter engagement on climate action-related themes in the building sector over various high-level policy events. This provides the extended results for Figures 3 and 4 in the main manuscript.

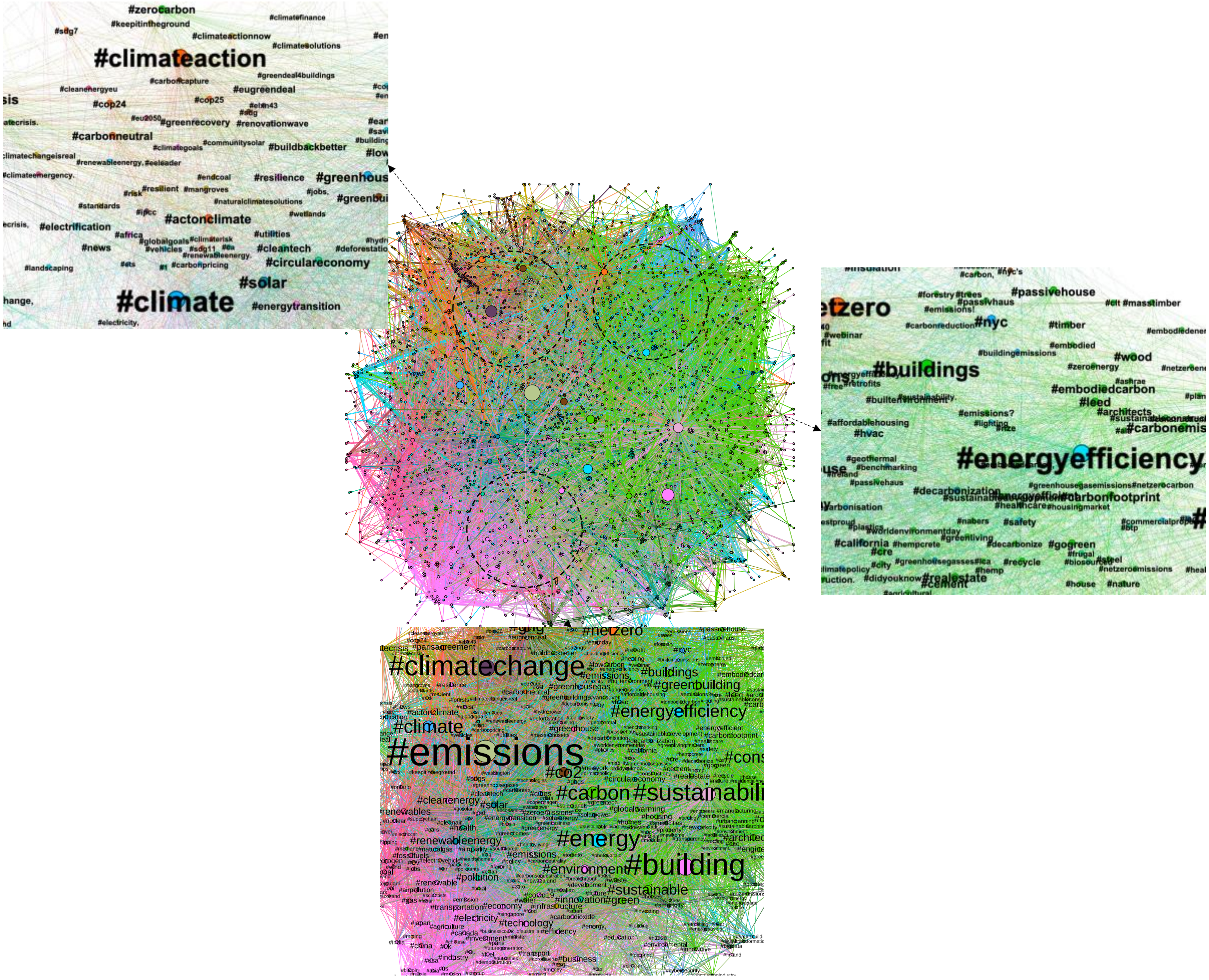

Fig A5: N3 network with hashtag clusters.

Fig A6 expands the N4(2021) network that specifically mentions the engagement around COP26 on Twitter. It is a complex network with several overlapping clusters denoting conversations on concrete, government and supply chain, hydrogen economy, net zero and cop26. This provides the extended results for Figure 5 in the main manuscript showing emergence of social and environmental justice themes in the Twitter discourse.

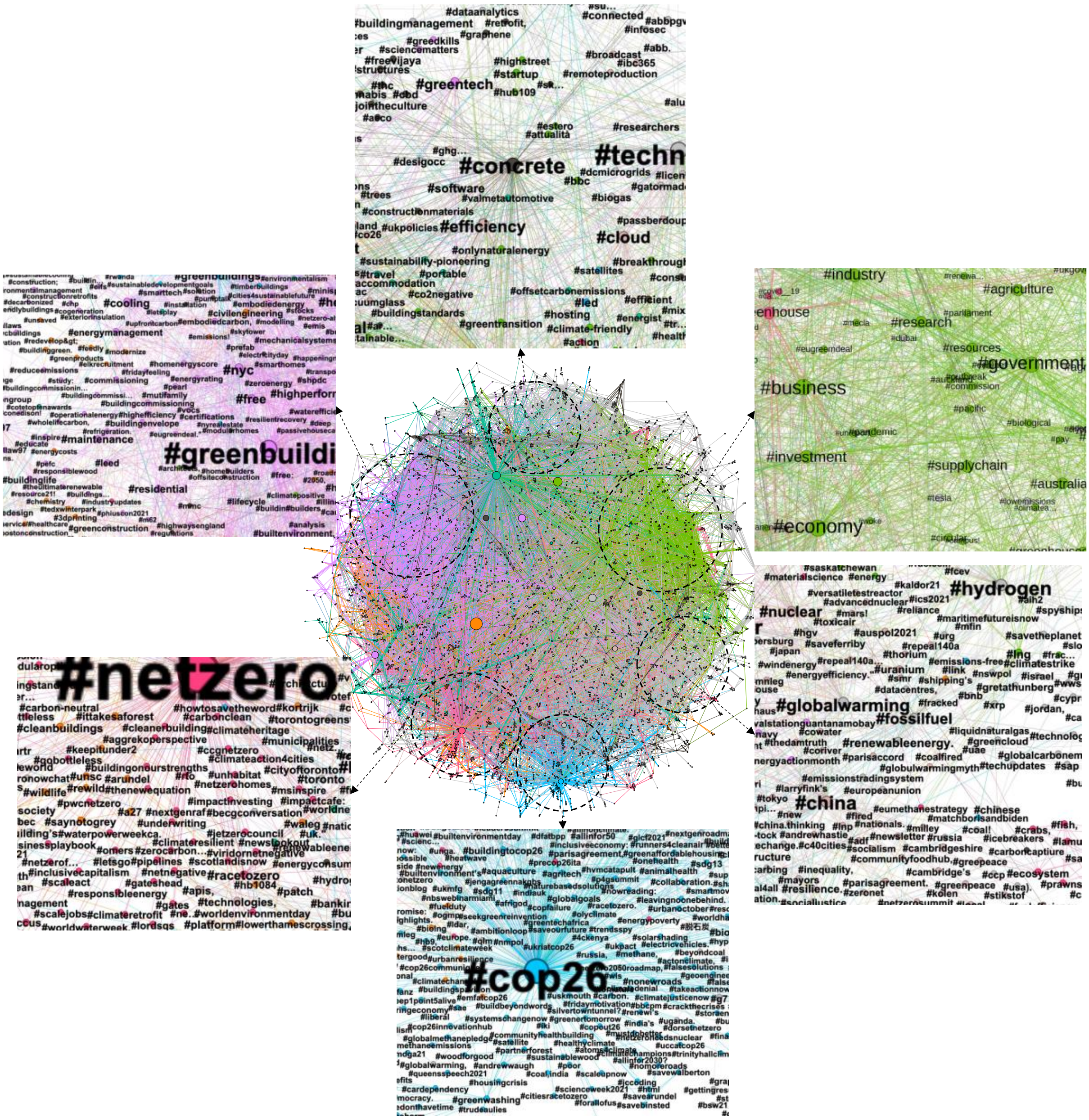

Fig A6: N4 (2021) network with hashtag clusters.

Section 2. Data characteristics

This section provides further information on the 256, 717 tweets, collected between 2009 – 2021, that are used in our analysis. Twitter provided this dataset using their latest v2 API which comprises of the entire sample of public tweets and retweets on #emission and #building (and its plural forms). Fig A7 shows the distribution of unique users (n = 188,096) who used with these hashtags. We derive these data characteristics using the metadata provided by Twitter during query generation. Key characteristics include user tweet count (Fig A8), user follower count (Fig A9), user following count (Fig A10) and retweet count (Fig A11). The data are shown in the boxplots and kernel density plots below.

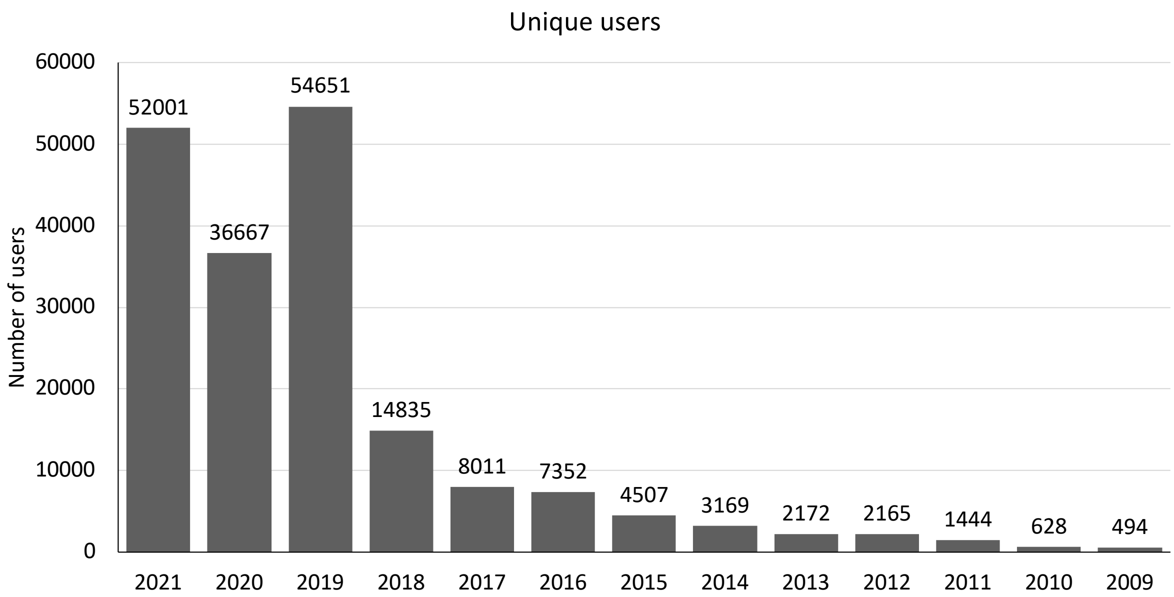

Fig A7 Unique Twitter users engaging with #emission(s) and #building(s) across 13 years. Total unique users = 188,096.

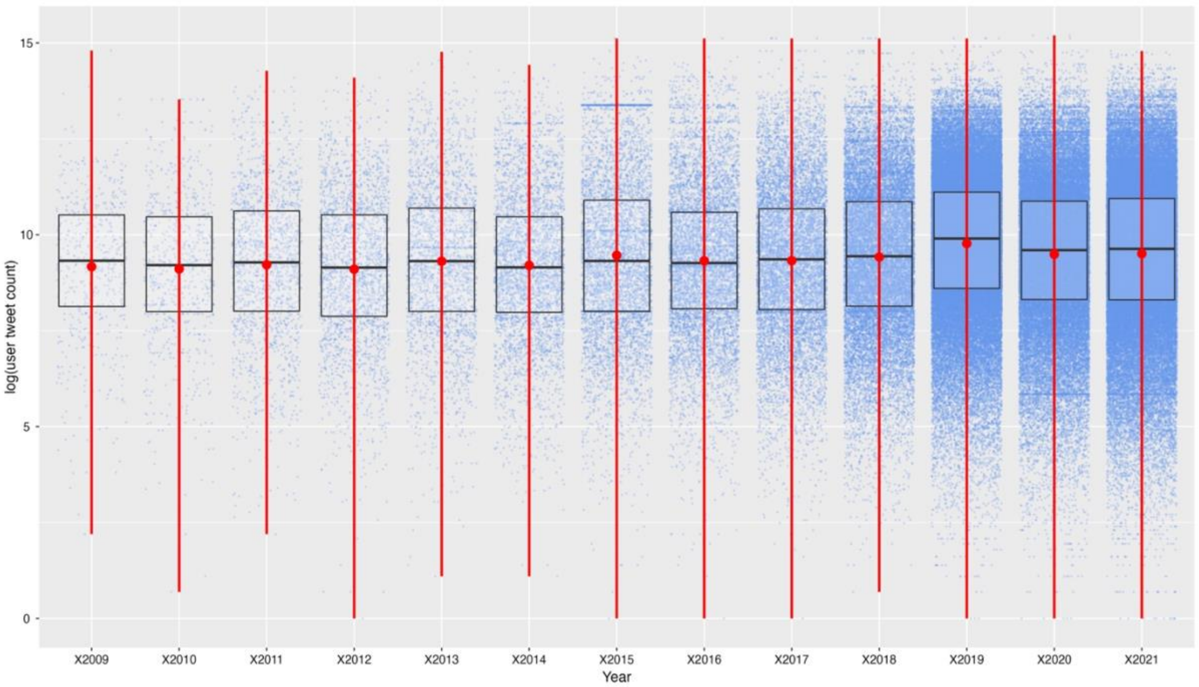

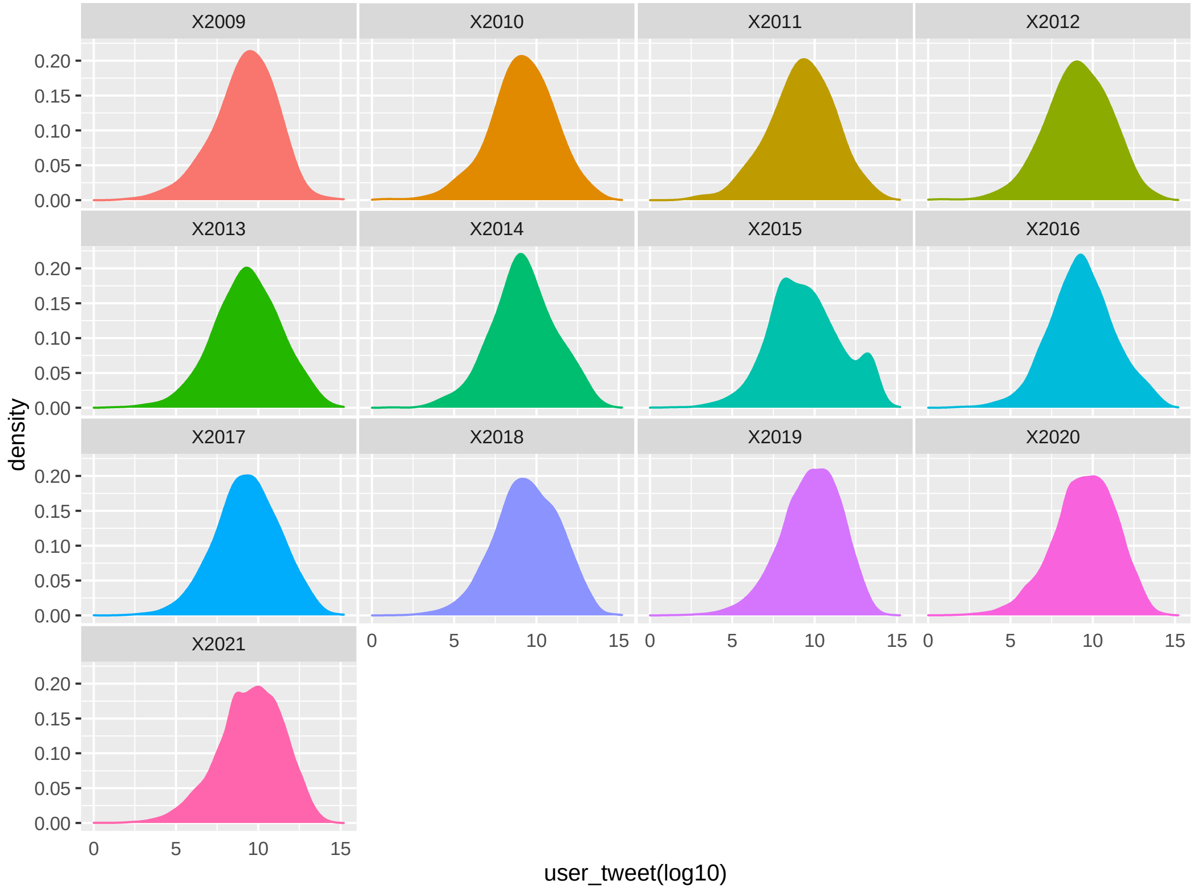

Fig A8 Boxplot and density plot of user tweet counts (2009 – 2021)

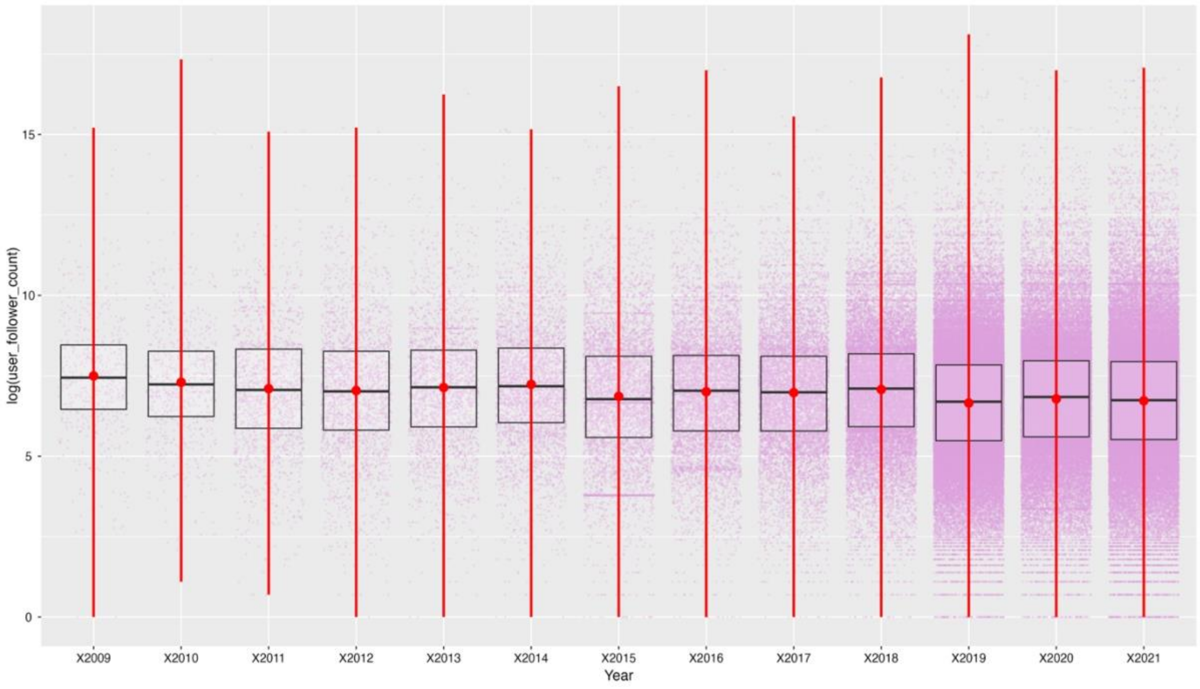

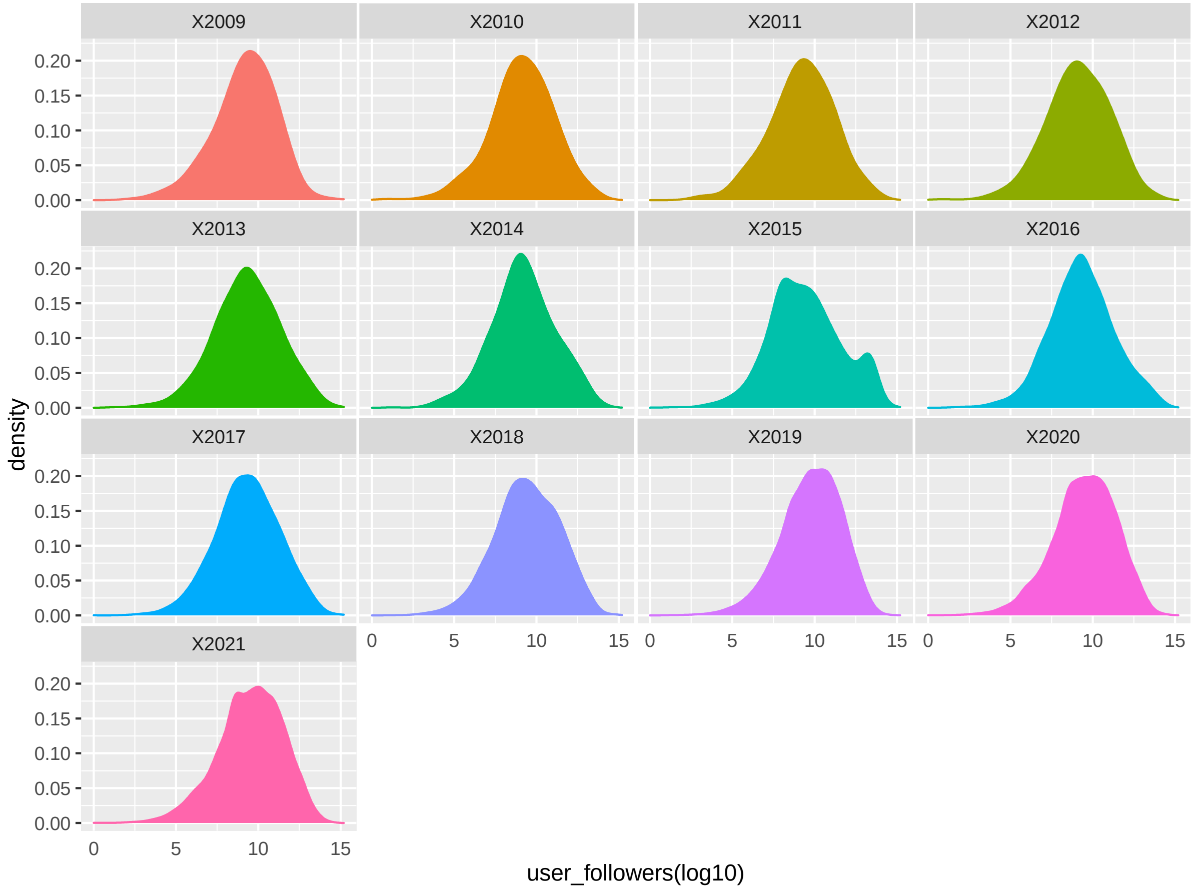

Fig A9 Boxplot and density plot of user follower counts (2009 – 2021)

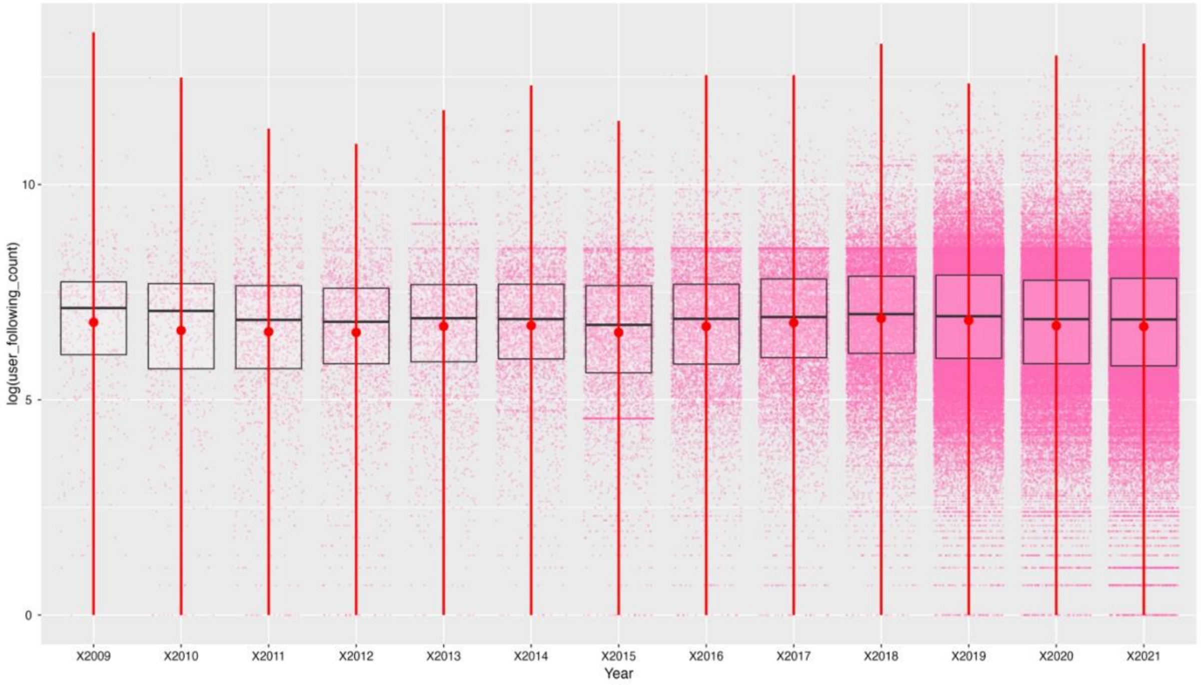

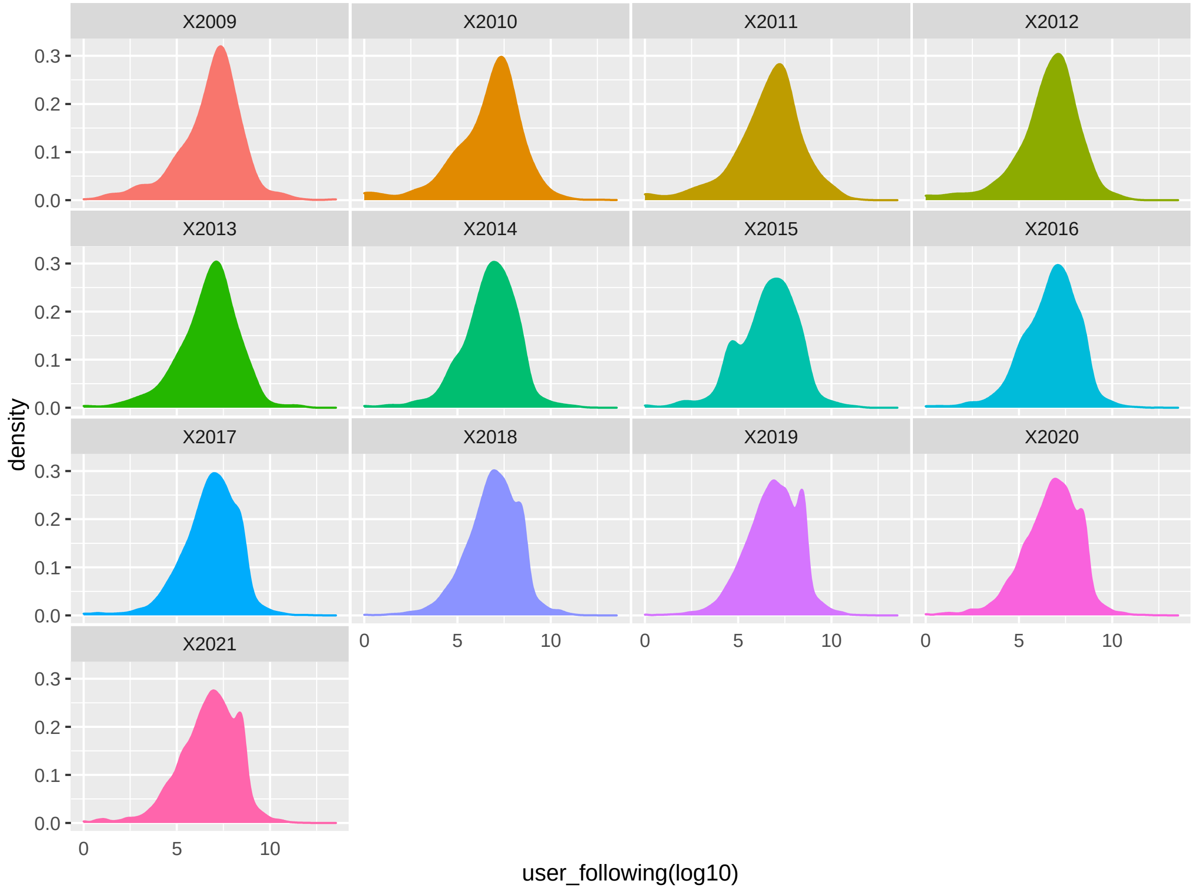

Fig A10 Boxplot and density plot of user following counts (2009 – 2021)

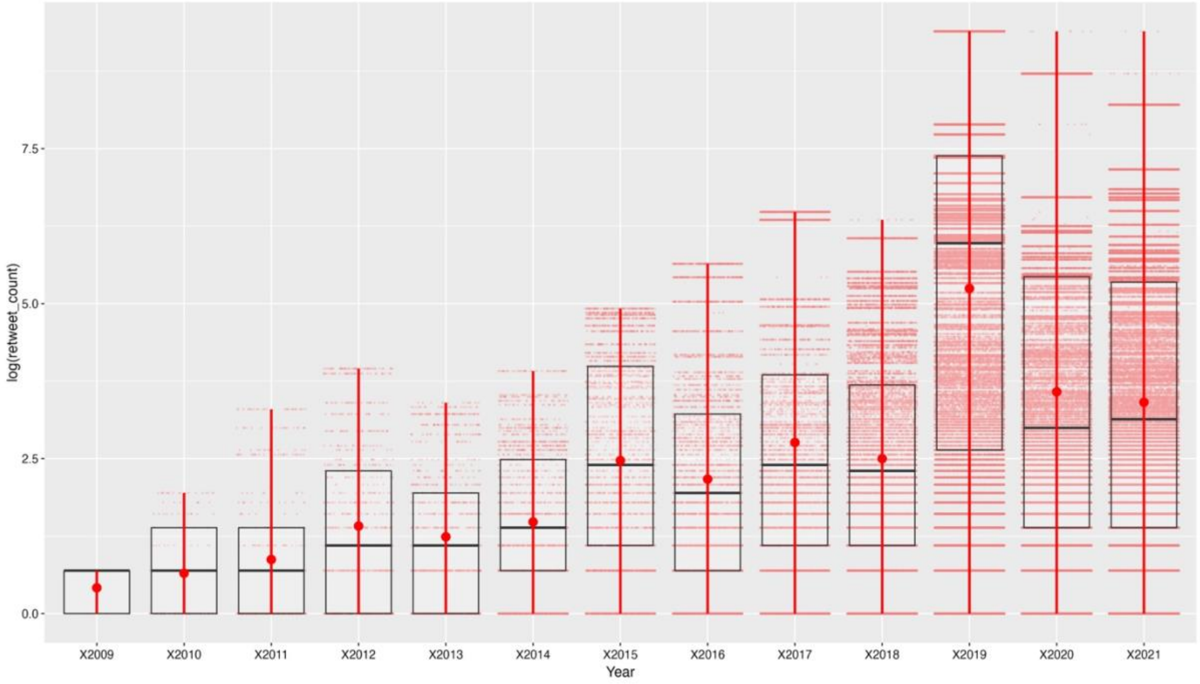

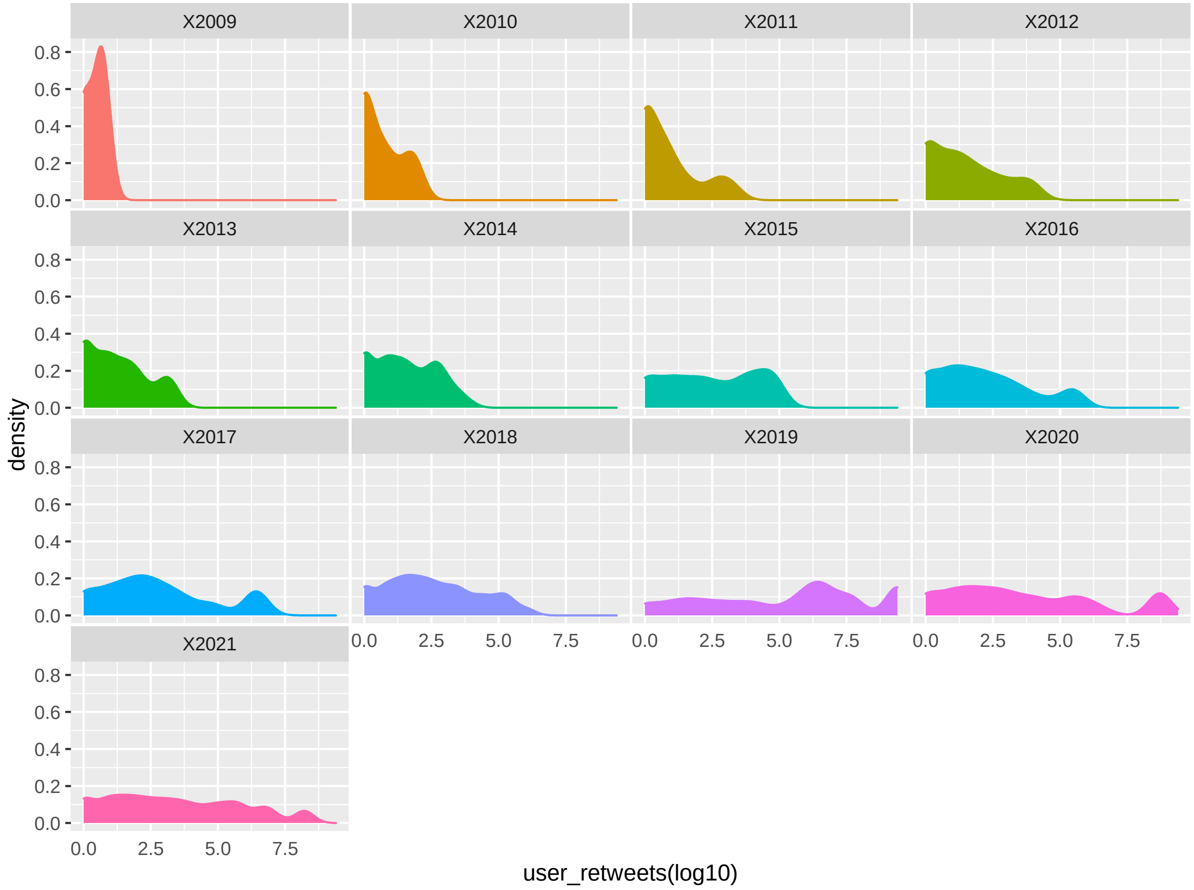

Fig A11 Boxplot and density plot of retweet counts (2009 – 2021)

References

Berglez, P. & Al-Saqaf, W. Extreme weather and climate change: Social media results, 2008–2017. *Environ. Hazards* 20, 382–399, DOI: 10.1080/17477891.2020.1829532 (2020)
